# Antimicrobial Nanozymes: Structure–Activity-Guided Design, Synergy, and Applications

**DOI:** 10.34133/research.1368

**Published:** 2026-07-28

**Authors:** Yuan Liu, Kaili Guo, Jingnan Shi, Xiaoxiao Guo, Shiwei Luo, Lizeng Gao, Xiyun Yan, Ye Yuan, Bing Jiang

**Affiliations:** ^1^ Nanozyme Laboratory in Zhongyuan, School of Basic Medical Sciences, Zhengzhou University, Zhengzhou 450001, China.; ^2^ Nanozyme Laboratory in Zhongyuan, Henan Academy of Innovations in Medical Science, Zhengzhou, Henan 451163, China.; ^3^ CAS Engineering Laboratory for Nanozyme, Key Laboratory of Protein and Peptide Pharmaceutical, Institute of Biophysics, Chinese Academy of Sciences, Beijing 100101, China.

## Abstract

Nanozymes are emerging as versatile antimicrobial agents to tackle drug-resistant infections and biofilm-associated persistence. In this review, we present an integrated framework that links natural enzyme-mediated host defense to the rational development of antimicrobial nanozymes, and summarize recent advances through a mechanism-materials-engineering-application pipeline. We first summarize representative bactericidal principles of natural enzymes to highlight bioinspired catalytic motifs relevant to nanozyme design. We then classify antibacterial nanozymes by catalytic reaction types, including oxidoreductase- and hydrolase-like activities, and by material platforms, highlighting structure–activity relationships that govern catalytic behavior and antimicrobial performance. Building on these mechanistic and structural insights, we summarize engineering-enhanced bactericidal modalities, including photothermal and photodynamic assistance, metal ion release, immune modulation, cascade catalysis, microenvironment-responsive regulation, and targeting, that help overcome constraints imposed by infectious microenvironments, such as hypoxia, limited hydrogen peroxide availability, elevated antioxidant levels, and biofilm barriers. Finally, we translate these principles into application-oriented guidance across interfaces ranging from abiotic surface protection, including antifouling and device coatings, to superficial and deep-seated infections, and we briefly discuss emerging nanozyme strategies for antifungal and antiviral interventions. Throughout, we emphasize translational considerations, such as activity benchmarking, biosafety evaluation, and scalable manufacturing, to support the development of clinically relevant antimicrobial nanozymes.

## Introduction

The worsening global crisis of antimicrobial resistance has posed unprecedented challenges to conventional anti-infective therapies. Rising drug resistance in bacteria, fungi, and viruses greatly hinders the management of common infectious diseases. World Health Organization (WHO) projections indicate that antimicrobial-resistant pathogens will lead to over 10 million deaths annually by 2050, ranking drug-resistant infections among the most severe global health threats [1].

Antimicrobial resistance arises from genetic mutation, horizontal gene transfer, and antibiotic-driven selection. Bacteria may acquire resistance genes from other organisms or spontaneous mutations. Additionally, intracellular bacteria and biofilm formation play key roles in the spread of such resistance [2]. Intracellular bacteria evade immune surveillance and antibiotic treatment, enabling them to survive and spread resistance genes to other pathogens. Biofilms, organized bacterial aggregates encased in a protective extracellular matrix, act as formidable physical barriers that impede antibiotic diffusion and prevent the effective infiltration of immune cells [3]. Additionally, the close contact between bacteria within biofilms facilitates gene exchange, promoting the spread of resistance genes and persistence of antibiotic-resistant strains. Therefore, developing new therapeutic approaches to combat drug resistance is crucial [4]. Studies have verified that natural enzymes exhibit outstanding catalytic efficiency and substrate specificity, enabling selective bacterial elimination with negligible cytotoxicity toward human cells via hydrolytic and oxidative pathways. However, critical limitations such as costly purification, low structural stability, and inconvenient storage hinder their broad clinical deployment. Drawing inspiration from the bactericidal pathways and catalytic active sites of natural enzymes, researchers have constructed nanozymes with enhanced stability and tunable catalytic behavior through biomimetic structural and functional design.

Nanozymes have recently gained recognition as a promising category of antimicrobial agents. Their unique capability to mimic the functions of natural enzymes, coupled with their tunable catalytic activity, enhanced stability, and tolerance to various environmental factors, positions them as highly valuable alternatives in combating infections [5]. These nanozymes demonstrate the ability to retain catalytic activity even in extreme conditions, highlighting their potential as effective therapeutic agents against bacterial, fungal, and viral infections.

The microbicidal, antifungal, and antiviral mechanisms of nanozymes fall into the following categories. First, these nanozymes directly eliminate microbes via enzyme-like activities. Some exhibit mono-enzymatic activity that generates reactive oxygen species (ROS) through catalytic oxidation; ROS disrupt microbial membranes, DNA, and proteins to suppress or eradicate pathogens. Others degrade extracellular DNA (eDNA) to disassemble biofilms or break down phospholipids to destroy bacterial envelopes. Second, several nanozymes carry multiple enzymatic functions and execute microbicidal activity via cascade catalytic reactions. Third, the intrinsic optical, acoustic, and thermal features of nanozyme components can amplify catalytic performance. Synergistic treatments including photodynamic, sonodynamic, and photothermal therapy further strengthen microbicidal potency. In addition, certain nanozymes bind to microbial surfaces to facilitate intracellular penetration of therapeutic agents and boost microbe-killing capacity. Finally, pristine or modified nanozymes display selective microbicidal capacity, which enables specific elimination of bacteria, fungi, or viruses under given conditions and lowers cytotoxicity toward host cells.

Although nanozyme synthesis, characterization, catalytic regulation, and biomedical applications have been extensively reviewed [6,7], most existing reviews are primarily organized according to material categories, catalytic activities, or specific application scenarios. For example, recent reviews have systematically summarized the structure–activity relationships, modification strategies, and theranostic applications of metal–organic framework (MOF)-based nanozymes [8], while Ghasemlou and coworkers [9] highlighted how surface chemistry and interface engineering regulate the catalytic performance, structural stability, and biointerfacial behavior of colloidal nanozymes. These studies have greatly advanced the understanding of nanozyme design and biomedical applications. However, a critical framework that integrates natural enzyme-mediated host defense, structure–activity relationships, infection microenvironment constraints, synergistic antimicrobial engineering, and translational considerations for antimicrobial nanozymes remains lacking.

In this review, we present a structure–activity-guided design framework for antimicrobial nanozymes by connecting natural enzyme-mediated host defense with the rational engineering of nanozyme catalytic systems. Rather than simply categorizing antimicrobial nanozymes according to catalytic activity or material type, we emphasize how structural parameters determine catalytic behavior and antimicrobial outcomes, how infection microenvironments shape nanozyme performance, and how synergistic engineering strategies can overcome these biological constraints. Finally, we discuss current challenges in activity benchmarking, biosafety, resistance evolution, and scalable manufacturing, with the aim of providing practical design principles for the development and clinical translation of next-generation antimicrobial nanozymes.

## Antibacterial Mechanisms of Natural Enzymes

In contrast to conventional antibiotics, natural enzymes stand out owing to their remarkable catalytic efficiency and exclusive substrate recognition. Apart from robust bactericidal capability, natural enzymes are intrinsically biocompatible and impose no damage to host cells.

Humans possess a robust innate host antimicrobial defense system driven by natural enzymatic cascades. For example, upon invasion by bacteria or other pathogens, macrophages engulf these foreign microbes to shield the host from infection. These immune cells are first recruited by pathogen-derived chemical signals, which facilitate pathogen binding to surface receptors on macrophages. The macrophages then internalize the invaders and sequester them within intracellular vesicles. Such vesicles fuse with cellular lysosomes, whose digestive enzymes destroy and break down captured pathogens. This pathogen clearance pathway relies on a coordinated multi-enzyme cascade. First, NADPH (reduced form of nicotinamide adenine dinucleotide phosphate) oxidase (OXD) catalyzes NADPH oxidation and oxygen reduction to produce superoxide anion radicals (·O_2_^−^). Superoxide dismutase (SOD) further converts these radicals into hydrogen peroxide (H_2_O_2_) and O_2_. Next, myeloperoxidase (MPO) catalyzes the reaction between H_2_O_2_ and chloride ions (Cl^−^) to yield hypochlorous acid (HClO), a potent oxidant that destroys microbial lipids, proteins, and nucleic acids. In parallel, acidic lysosomal environments trigger ion-mediated Fenton reactions to generate hydroxyl radicals (·OH), the most reactive form of ROS. These unselective radicals fragment bacterial DNA, degrade membrane lipids, and disrupt ribosomal architectures, enabling rapid bacterial clearance.

Beyond the cascade catalytic microbicidal pathways inside phagocytes, other natural enzymes also exhibit potent microbe-eliminating activity. Among proteases, studies demonstrate that Bacillus subtilisin eliminates biofilms produced by *Pseudomonas*, *Xanthomonas*, and *Bacillus* strains and blocks coaggregation between *Nocardia*, oral streptococci, and *Propionibacterium* via hydrolytic degradation of adhesin proteins. Lysostaphin cleaves the pentaglycine cross-bridges of staphylococcal cell walls between the third and fourth glycine residues, triggering cell wall lysis and eradicating pathogens including meat-fermenting staphylococci, iatrogenic *Staphylococcus aureus*, *Staphylococcus epidermidis*, *Staphylococcus saprophyticus*, and *Staphylococcus xylosus*. Bacteriophage lysozymes are viral proteins that enable phages to invade host bacteria during infection and exit post-replication. Such lytic enzymes also exert bactericidal activity against phage host strains.

As a polysaccharide hydrolase, lysozyme mainly targets Gram-positive bacteria; it also damages some Gram-negative strains by breaking β-1,4-glycosidic linkages in bacterial cell walls. Substantial efforts are still devoted to expanding its practical applications. Dispersin B, amylase, and pectin methylesterase are native hydrolases capable of eradicating *S. aureus* and *Escherichia coli*. They also display potent antibiofilm activity against *S. aureus* biofilm matrices. Alginate lyase likewise carries hydrolytic capacity. Research confirms that it severs β-glycosidic linkages in bacterial alginate polymers to eliminate *Pseudomonas aeruginosa*. Furthermore, chitinases break glycosidic bonds in fungal cell wall chitin and thereby deliver prominent antifungal activity.

Lactonase blocks bacterial quorum sensing (QS) by degrading acyl-homoserine lactones (AHLs), thereby inhibiting the secretion of virulence factors and biofilm formation [10]. In QS, a bacterial cell-to-cell communication system, bacteria release and respond to signaling molecules to orchestrate group behaviors, including gene transfer, secretion of virulence factors, sporulation, and biofilm formation. Anti-quorum sensing enzymes, such as lactonase, can hydrolyze ester bonds in AHL rings. This blocks AHL–receptor binding to transcriptional regulators and suppresses bacterial pathogenicity. Researchers have adopted this strategy to target *P. aeruginosa*.

Glucose oxidase drives H_2_O_2_ generation and d-gluconic acid accumulation. Studies have validated its antimicrobial potency against *S. aureus*, *Bacillus cereus*, *Clostridium perfringens*, *Salmonella infantis*, *Campylobacter jejuni*, *Yersinia enterocolitica*, and *Listeria monocytogenes*. Glucooligosaccharide dehydrogenase utilizes diverse oligosaccharides as electron donors to reduce quinones or generate H_2_O_2_, and it eradicates pathogens including *S. aureus*, *Bacillus subtilis*, *Xanthomonas*, and *E. coli*. Furthermore, haloperoxidases (HPOs) exhibit broad-spectrum activity against both Gram-positive and Gram-negative bacterial strains [11]. Its antimicrobial effect is achieved through the conversion of halides or pseudohalides into stronger antimicrobial agents via H_2_O_2_-dependent oxidation.

Natural enzymes feature high catalytic efficiency, strict substrate specificity, and eco-friendly antibacterial performance, making them promising supplements to conventional antibiotics. They stand out particularly for fighting drug-resistant bacterial strains. Nevertheless, the intrinsic shortcomings of natural enzymes make large-scale substitution of antibiotics unfeasible. First, natural enzymes only possess a narrow antibacterial spectrum; most display strong activity against Gram-positive bacteria yet weak efficacy toward Gram-negative species, owing to the protective outer membrane barrier. Second, complicated extraction and purification procedures drive up their mass-production costs relative to antibiotics. Hence, more research is required to design more efficient, practically viable microbicidal strategies.

Taken together, natural antimicrobial enzymes provide not only biological examples of pathogen killing but also transferable design principles for antimicrobial nanozymes. The oxidative cascade in phagolysosomes, represented by NADPH OXD, SOD, MPO, and Fenton-type chemistry, inspires peroxidase (POD)-, OXD-, SOD-, catalase (CAT)-, and HPO-like nanozymes that regulate ROS or hypohalous acid (HOX) generation. Cell wall- and membrane-cleaving enzymes, including lysozyme, lysostaphin, and phospholipases, motivate hydrolase-like nanozymes that weaken bacterial envelopes or directly hydrolyze phospholipid membranes. Deoxyribonuclease (DNase)-like activity provides a rationale for designing nanozymes that degrade eDNA and destabilize biofilm matrices. Lactonase-mediated quorum-sensing disruption suggests that nanozymes can also be engineered to attenuate virulence and biofilm formation rather than only kill bacteria directly.glucose oxidase (GOx)- and OXD-related reactions further inspire cascade nanozyme systems that self-supply hydrogen peroxide, acidify local microenvironments, and amplify downstream POD-like catalysis.

Therefore, the transition from natural enzymes to nanozymes should be understood as a shift from mimicking individual enzyme functions to engineering modular antimicrobial catalytic systems. Nanozymes do not fully reproduce natural enzyme pockets; instead, they replicate, simplify, or substitute key catalytic features, including metal-centered redox cycling, active site coordination, substrate adsorption, confined reaction microenvironments, and cascade coupling. This design logic provides the foundation for the nanozyme mechanisms and structure–activity relationships discussed in the following sections.

From a biomimetic perspective, natural enzymes achieve catalytic efficiency and substrate specificity through highly organized active site microenvironments. These microenvironments usually consist of metal centers or catalytic residues, well-defined coordination structures, hydrogen-bonding networks, hydrophobic or charged pockets, and spatial confinement that orient substrates and stabilize reaction intermediates. Antimicrobial nanozymes do not completely reproduce such proteinaceous pockets. Instead, they replicate, simplify, or substitute the key catalytic elements of natural enzymes through atomically dispersed metal sites, metal–ligand coordination motifs, surface defects, porous confinement, surface ligands, and interfacial microenvironments. This simplified biomimetic strategy sacrifices part of the substrate specificity of natural enzymes but improves structural robustness, environmental tolerance, catalytic tunability, and multifunctional integration. Therefore, the theoretical basis of antimicrobial nanozyme design lies not in copying natural enzymes as a whole but in extracting transferable catalytic microenvironments and reconstructing them using stable and engineerable nanomaterial platforms.

## Antibacterial Mechanisms of Nanozymes

**Fig. 1.**
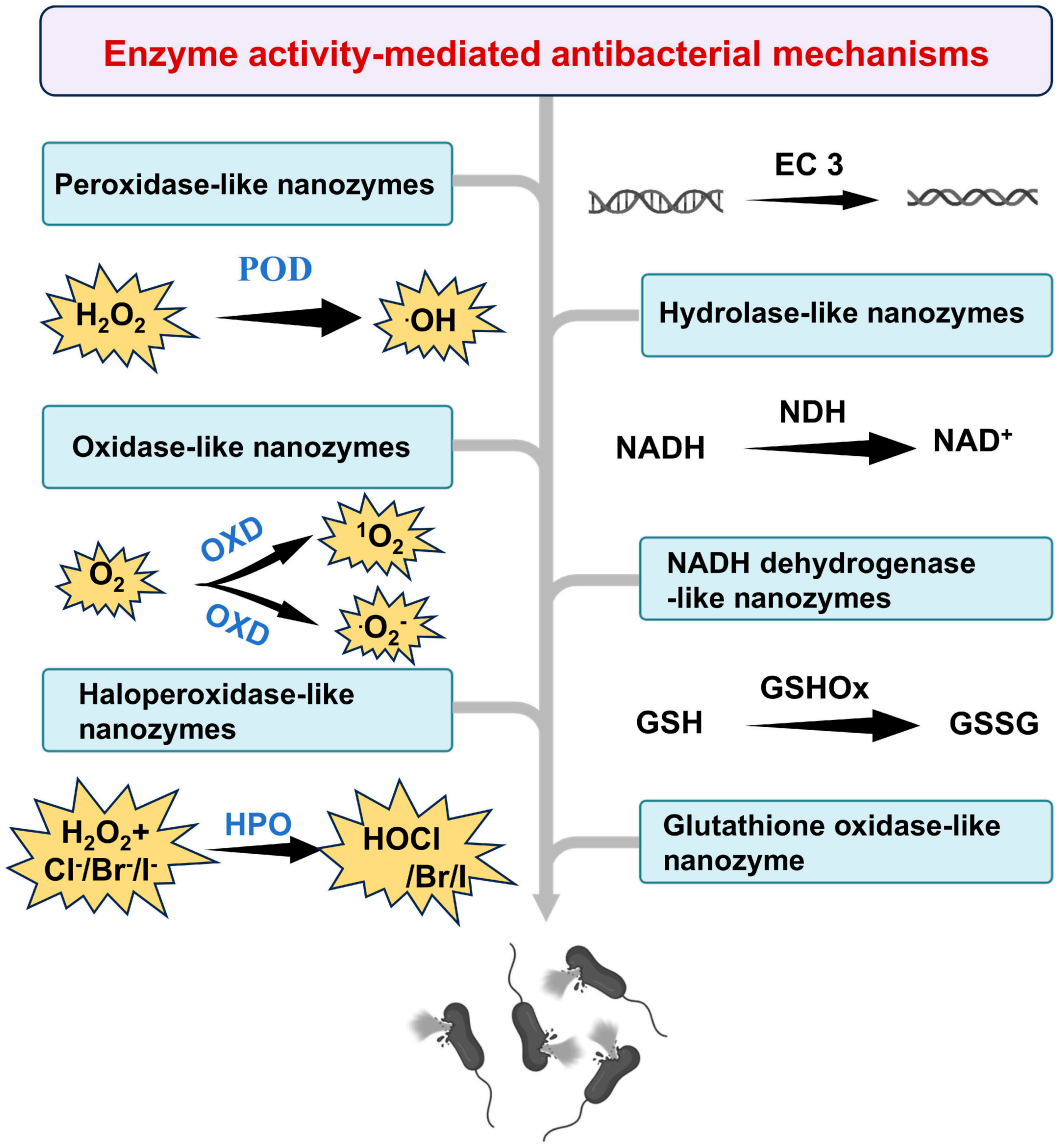
Representative antibacterial catalytic mechanisms of nanozymes.

Nanozymes are functional nanomaterials capable of replicating the catalytic behaviors of natural enzymes. Their design evolution has shifted from functional simulation to structural imitation, and progressed toward multi-functional integration within biomimetic catalytic platforms [12]. Early nanozymes were mostly discovered serendipitously. They merely replicated the catalytic functions of natural enzymes with crude structural design, and thus failed to overcome the limitation of poor substrate specificity. With advances in characterization techniques, researchers have shifted their focus to mimicking the active sites, spatial microenvironments, and 3-dimensional (3D) conformations of natural enzymes [13], which greatly boosts catalytic specificity and efficiency. Considering the requirements of practical applications, single-function catalysts cannot accommodate complex physiological microenvironments. Accordingly, next-generation biomimetic catalytic systems are engineered for multifunctional integration. At present, rational design of high-performance nanozymes primarily depends on atomic-scale regulation of catalytic metal sites to replicate the active pockets of natural enzymes. Nanozymes have successfully reproduced the catalytic behaviors of diverse natural enzymes, as summarized in Table 1. For instance, focusing on POD-like nanozymes, Jiang and colleagues [14] constructed the HCFe nanozyme. This material adopts a hemin–cysteine–Fe moiety as its catalytic core, which imitates the active centers of natural horseradish peroxidase (HRP). The HCFe nanozyme exhibits outstanding POD-like catalytic activity. Fan et al. [15] have discovered nanozymes that can surpass natural HRP, which is a milestone indicating that nanozymes have reached a creative level from biomimetic mode. For CAT-like nanozymes, Ji and colleagues [16] fabricated an Fe_2_-SAzyme with prominent catalase-mimetic activity. In the Fe_2_-SAzyme structure, iron single atoms are immobilized on a nitrogen-doped carbon substrate to form an Fe-N_4_ coordination configuration, which mimics the heme domain of natural catalase. The synergy between the dual iron active sites within the material grants it predominant CAT-like performance, while its intrinsic POD-like capacity is largely inhibited. For OXD-like nanozymes, Yu et al. [17] synthesized peptide-copper coassemblies with laccase-mimetic activity. This work rationally designed peptide–metal assemblies to replicate the intermediate state (IS) of laccase active centers, precisely reconstructing the trinuclear copper cluster (T2–T3) and its coordination microenvironment critical for O_2_ reduction and electron transport. For SOD-like nanozymes, the Zhang group [18] rationally designed and fabricated CuZnHis(L) supramolecular assemblies. This biomimetic material replicates the Cu^2+^ active site of natural Cu/Zn-SOD and modulates its coordination configuration: The native 4-histidine (Cu-N_4_) coordination is transformed into an amine-carboxylate-bis-imidazole (Cu-N_2_O_2_) coordination within Cu/Zn/histidine assemblies. However, disparities in inherent architectures explain the gaps in catalytic performance, structural robustness, and substrate selectivity between native enzymes and nanozymes. Natural enzymes depend on accurate protein folding to build unique pocket surroundings around catalytic cores, which guarantee high turnover efficiency and stringent substrate identification. Even so, protein skeletons readily denature under harsh surroundings, impairing structural durability. As inorganic biomimetic catalysts, nanozymes possess rigid frameworks and uncomplicated surface pockets. Although their substrate recognition capacity is weaker, they exhibit outstanding tolerance to extreme environments. Benefiting from numerous exposed catalytic sites, nanozymes can achieve comparable catalytic performance suitable for practical applications. At present, nanozymes have been extensively explored in various research directions, especially antibacterial therapy. Based on catalytic classification, nanozymes fall into 2 major groups: oxidoreductase mimics and hydrolase mimics. Most nanozymes display oxidoreductase-mimetic performance, covering SOD-, POD-, CAT-, and OXD-like activities. In contrast, only a small fraction reproduce catalytic behaviors similar to hydrolases or other enzyme families. By rationally tuning enzyme-mimetic properties, nanomaterials with customized functions can be fabricated. Existing studies on hydrolytic nanozymes mainly center on antibiofilm scenarios, while oxidoreductase-like nanozymes realize bactericidal effects chiefly via ROS production. The catalytic pathways of various bactericidal nanozymes are summarized in Fig. 1.

**Table 1.** The summarization of antibacterial mechanisms of nanozymes and natural enzymes

Catalytic types	Natural enzymes	Catalytic active center	Catalytic mechanisms	Natural enzyme bactericidal mechanism	Nanozymes	Nanozyme bactericidal mechanisms
ROS generation	Peroxidase	Heme iron: Fe^3+^/Fe^2+^	Utilizes H_2_O_2_ to oxidize substrates while undergoing valence cycling	Catalyzes the generation of ·OH, causing oxidative damage to bacterial lipids, proteins, and DNA	Carbon-based nanozymes, noble metal-based nanozymes, single-atom nanozymes	Mimics peroxidase activity, decomposing H_2_O_2_ to generate ·OH
	Haloperoxidase	Heme iron: Fe^3+^/Fe^2+^	Utilizes H_2_O_2_ to oxidize halide ions (Cl^−^, Br^−^, I^−^) to hypohalous acids (HOX)	Produces HOCl/HOBr, which are strongly oxidizing, long-lived, and have good penetration, widely damaging bacterial membrane structures and biomacromolecules, especially effective in disrupting biofilms		Mimics haloperoxidase activity to produce HOCl/HOBr
	Oxidase	Molybdenum cofactor, FAD, iron-sulfur clusters	Catalyzes substrate dehydrogenation, directly transferring electrons to O_2_, and then generate H_2_O_2_ or ·O_2_^−^	Produces H_2_O_2_ or ·O_2_^−^, inducing oxidative stress		Mimics oxidase activity, using O_2_ as an electron acceptor to directly catalyze the generation of H_2_O_2_/·O_2_^−^
Hydrolysis of biofilm/bacterial cell wall	Phospholipase	Catalytic triad or calcium-binding site (type-dependent)	Hydrolyzes the ester bonds in phospholipid molecules, generating lysophospholipids and fatty acids	Directly disrupts the integrity of the bacterial cell membrane’s phospholipid bilayer, causing leakage of cellular contents and lysis	Noble metal-based nanozymes, metal organic frameworks nanozymes	Mimics phospholipase activity, directly hydrolyzing membrane phospholipids
	Nuclease	Metal ions (Mg^2+^, Ca^2+^) and conserved acidic amino acid residues	Hydrolyzes phosphodiester bonds, cleaving DNA or RNA strands	1. Direct killing: Degrades the bacterium’s genetic material (DNA/RNA) upon invasion, preventing replication and protein expression. 2. Clearing eDNA: Degrades eDNA		Mimics nuclease activity to cleave bacterial DNA/RNA under specific conditions
Metabolic regulation	NADH oxidase	FAD/FMN, metal Ions	Catalyzes the oxidation of reductive coenzymes like NADH, depleting intracellular reducing power	Depletes bacterial energy sources: oxidizes NADH, disrupting bacterial energy metabolism and redox homeostasis, inhibiting growth	Polymeric nanozymes, composite nanozymes	Mimics oxidoreductase activity by depleting key metabolites like NADH, thereby disrupting their redox balance

Notably, among POD- and OXD-like nanozymes for antibacterial therapy, a ROS-mediated bactericidal mechanism represents the most well-documented antibacterial pathway. Excess intracellular ROS initiate lipid peroxidation and membrane depolarization, destroying the structural integrity of bacterial envelopes. Meanwhile, ROS oxidize intracellular proteins and metabolic enzymes to suppress energy metabolism, and inflict irreversible DNA damage, ultimately arresting bacterial proliferation and triggering cell lysis. Nevertheless, ROS generation is not the only killing route of such nanomaterials. Growing evidence confirms that multiple non-ROS-dependent pathways also contribute greatly to pathogen clearance, including membrane depolarization, metabolic suppression, ferroptosis-like bacterial death, disrupted ionic balance, and immune modulation. Nanozymes alter bacterial transmembrane potential through physical contact or electrostatic attraction, raising membrane permeability and triggering cation leakage, which collapses membrane function and causes bacterial death. Metabolic suppression hinders bacterial nucleic acid replication, protein synthesis, and energy turnover to restrain pathogen propagation. Ferroptosis-like bacterial death features intracellular iron overload, glutathione (GSH) depletion, lipid peroxidation of membrane polyunsaturated fatty acids, and deactivated antioxidant defenses. This iron-dependent programmed cell death delivers powerful bactericidal efficacy, particularly against drug-resistant strains [19,20]. These mechanisms often coexist with ROS generation in practical antimicrobial systems, indicating that nanozyme-mediated microbial clearance should be understood as a multi-pathway process rather than a single oxidative-damage event.

### Oxidoreductase-like nanozymes

Redox nanozymes drive redox reactions and accelerate intermolecular electron transfer. The category covers POD-, CAT-, OXD-, and SOD-like nanozymes. Among them, POD- and OXD-mimetic nanomaterials generate ROS, while CAT- and SOD-like counterparts scavenge ROS. Relying on their diverse catalytic behaviors, these redox nanozymes tune biological redox homeostasis. For antibacterial applications, POD- and OXD-like nanozymes eliminate pathogenic bacteria via massive ROS generation, and ROS yields are tightly correlated with their catalytic performance [21]. To construct nanozymes with strong ROS-generating capacity, researchers proposed a universal structure–activity correlation between natural enzymes and nanozymes. They further translated natural enzymatic catalytic mechanisms into rational nanozyme design principles, as summarized in Table 1. Following this bioinspired route, scientists have fabricated diverse nanozymes by reproducing the precise catalytic pocket structures of natural enzymes, their catalytic cycle conformational shifts, and unique biological templates derived from organisms. For instance, extensive research has focused on engineering nanozymes with tailored binding domains and catalytic cores. Such designs aim to reconstruct the catalytic microenvironment of native enzymes, boost catalytic turnover, and yield high-performance nanozymes with outstanding catalytic capacity. These materials achieve catalytic performance comparable to, or even superior to, that of natural enzymes. Moreover, incorporation of core catalytic motifs and structural signatures from natural enzymes enables the engineering of nanozymes with outstanding activity and substrate selectivity. For example, Wang and colleagues [22] constructed an AuPtCu@HRP-hapten nanozyme. Because of plentiful polymetallic catalytic centers, the hybrid displays outstanding POD-like activity. Via specific antigen–antibody binding, the nanozyme holds great potential for antibiotic residue sensing. The bioinspired concept is expected to garner widespread attention in both basic research and practical applications. It also provides deeper insights into the rational engineering of nanozymes, enabling fine-tuning of their catalytic performance [23].

#### POD-like nanozymes

POD-like nanozymes can act on various chromogenic substrates including TMB (3,3′,5,5′-tetramethylbenzidine), DAB (diaminobenzidine), and OPD (o-phenylenediamine) to produce distinct colorimetric signals. As dual-substrate catalytic systems, this category of materials can catalyze the oxidant substrate H_2_O_2_ to yield massive oxidative free radicals, which rapidly oxidize the chromogenic donor (TMB). Depending on the selected electron donor, POD-like nanozymes reproduce the catalytic behaviors of multiple enzymes such as HPO, MPO, and GPx (glutathione peroxidase). For instance, MOF-modulated Fe_3_O_4_-Au hybrids possess inherent POD-like activity. In the presence of low-dose H_2_O_2_ (0.97 mM), high loading of these hybrids generates abundant ·OH radicals, which greatly boost in vitro bactericidal efficacy and facilitate in vivo wound repair [24–26]. Similarly, ultrasmall ceria-cluster nanozymes display HPO-like activity. They catalyze the H_2_O_2_-mediated oxidation of bromide to generate bactericidal hypobromous acid, which suppresses colonization by multidrug-resistant bacterial strains (*E. coli* and *S. aureus*). This property makes them suitable as bioactive coating additives to restrain biofilm formation in marine environments [27,28].

#### Oxidase-like nanozymes

Oxidase-like nanozymes are a class of artificial enzymes that operate independent of H_2_O_2_. Instead, they abstract hydrogen using molecular oxygen as the electron acceptor and directly drive oxidation of hydrogen-donor chromogens like TMB. For antibacterial scenarios, their bactericidal capacity mainly arises from strongly oxidative singlet oxygen (^1^O_2_) and ·O_2_^−^, produced via OXD-catalyzed reduction of molecular oxygen. Studies have proven that Aux/Fe nanozyme can activate O_2_ and facilitate O–O bond cleavage. The material delivers exceptional OXD catalytic selectivity and performance, endowing it with great promise for acetylcholinesterase (AChE) sensing [29,30]. Furthermore, GSH is crucial for sustaining bacterial survival. Glutathione oxidase (GSHOx) can oxidize GSH to glutathione disulfide (GSSG), which results in a decreased GSH/GSSG ratio. Under oxidative stress, bacteria struggle to maintain normal viability.

#### NADH dehydrogenase-like nanozymes

Nicotinamide adenine dinucleotide (NADH) participates in diverse physiological processes to modulate cellular metabolism and energy production. In both eukaryotes and prokaryotes, NADH dehydrogenase binds NADH and transfers 2 high-energy electrons to its cofactors, oxidizing NADH to NAD^+^ [nicotinamide adenine dinucleotide (oxidized form)] simultaneously. Severe NADH depletion suppresses adenosine triphosphate (ATP) biosynthesis and cuts off cellular energy replenishment. Studies confirm that 808-nm near-infrared (NIR) light endows Cu-N-GQDs@Ru-NO nanozymes with NADH dehydrogenase-mimetic (NDH) activity, and the nanozyme mediates the oxidation of NADH into NAD^+^. This perturbs intracellular bacterial redox homeostasis and ultimately triggers bacterial death [31].

Despite the intrinsic merits of OXD-like nanozymes, excess ROS inevitably cause off-target tissue injury. Moreover, their long-term biocompatibility and toxicological characteristics remain to be thoroughly explored.

### Hydrolase-like nanozymes for antibiofilm applications

ROS-generating nanozymes can efficiently eliminate planktonic bacteria, but their activity against mature biofilms is often limited because planktonic killing and biofilm eradication represent fundamentally different antimicrobial scenarios. Conventional antibacterial assays mainly evaluate unprotected, metabolically active free-floating bacteria, and their efficacy is commonly quantified by minimum inhibitory concentration (MIC), minimum bactericidal concentration (MBC), time–kill curves, and colony-counting assays. In contrast, biofilms are compact 3D microbial communities embedded in extracellular polymeric substances (EPSs), which contain polysaccharides, proteins, lipids, and eDNA. This matrix creates diffusion barriers, heterogeneous pH and oxygen gradients, antioxidant buffering, and nutrient limitation, while embedded bacteria often adopt dormant or slow-growing states.

**Fig. 2.**
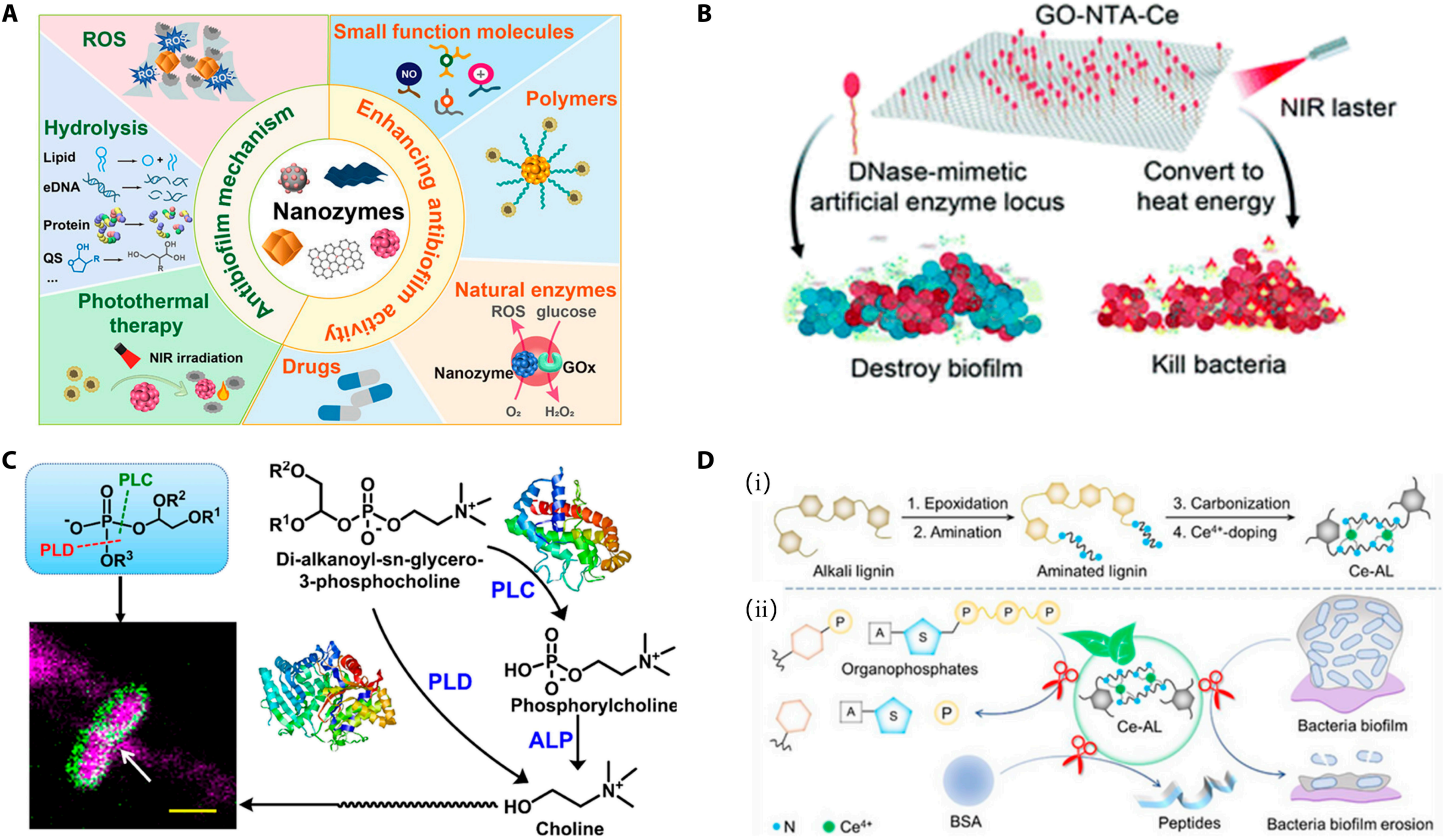
Hydrolase-like nanozymes for antibacterial applications. (A) Recent advances in nanozymes and their composites for antibiofilm applications [32]. Copyright 2025, American Chemical Society. (B) Schematic diagram of GO-NTA-Ce DNase-mimicking nanozyme for eradicating drug-resistant bacterial biofilms [33]. Copyright 2022, Royal Society of Chemistry. (C) Nanoceria-based phospholipase-mimetic cell membrane disruptive antibiofilm agents [34]. Copyright 2020, American Chemical Society. (D) (i) Schematic illustration of the preparation of Ce-AL. (ii) Application of Ce-AL for combating the *E. coli* and *S. aureus* biofilms [35]. Copyright 2026, Wiley-VCH Verlag.

Therefore, antibiofilm activity should be evaluated using biofilm-specific parameters, including minimum biofilm inhibitory concentration (MBIC), minimum biofilm eradication concentration (MBEC), biomass reduction, crystal violet staining, confocal laser scanning microscopy (CLSM), EPS degradation, eDNA degradation, biofilm penetration, and persister cell clearance. Nanozymes with strong activity against planktonic bacteria may still fail to eradicate mature biofilms if they cannot penetrate or dismantle the EPS matrix. Accordingly, effective biofilm removal usually requires multiple coordinated actions, including EPS degradation, destruction of biofilm architecture, quorum-sensing suppression, exposure of embedded bacteria, and subsequent bacterial inactivation (Fig. 2A) [32].

Compared with POD-mediated biofilm disruption, hydrolase-involved antibiofilm processes are more attractive. EPS function as the primary structural scaffold of biofilms. eDNA acts as a critical mediator that stabilizes the EPS network, preserves the 3D architecture of biofilms, and promotes bacterial flocculation; it also mediates the emergence of bacterial drug tolerance. Ji and colleagues [33] fabricated a graphene oxide (GO)–nitrilotriacetic acid (NTA)–Ce nanozyme with DNase-mimetic activity. The hybrid can trigger phosphodiester bond breakage and efficiently decompose biofilm-embedded eDNA. Once eDNA undergoes hydrolytic breakdown, the whole biofilm architecture loses structural support and collapses stepwise. Bacteria trapped inside the EPS matrix are thereby fully exposed. Afterward, the GO-NTA-Ce hybrid achieves thorough bacterial eradication through membrane destruction and photothermal ablation (Fig. 2B). Mugesh and colleagues [34] fabricated ceria-based nanozymes with phospholipase-like activity. Owing to their tiny particle size and positive surface charge, the nanozymes can permeate EPS to reach embedded bacteria. They cleave phospholipid ester linkages within bacterial membranes, triggering membrane disruption, leakage of cytoplasmic contents, and eventual bacterial death (Fig. 2C). Chen and colleagues [35] fabricated Ce-AL nanozymes with thermally responsive hydrolase-like activity. Upon thermal stimulation, their catalytic activity rises dramatically. The nanozymes efficiently hydrolyze eDNA and EPS, fully dismantling the cross-linked biofilm matrix. Meanwhile, the produced ROS trigger oxidative stress injury to bacteria, granting the hybrid outstanding antibiofilm capacity (Fig. 2D). A major strategy for biofilm removal is to expose embedded bacteria so that they can be directly accessed by antibacterial agents, which is a key research direction in this field.

## Structure–Activity-Guided Design of Antimicrobial Nanozymes

The antimicrobial performance of nanozymes is ultimately governed not only by their material composition but also by how structural parameters regulate catalytic behavior under biologically relevant conditions. Therefore, understanding structure–activity relationships is essential for establishing a rational design framework for antimicrobial nanozymes. Structural features, including particle size, morphology, oxidation state, defects, surface chemistry, and interfacial architecture, collectively determine substrate adsorption, active site accessibility, electron transfer, reaction pathways, and catalytic selectivity, thereby influencing reactive species generation, bacterial interactions, biofilm disruption, immune modulation, and biosafety.

Mechanistically, these structure–activity relationships should be understood as a multistep chain rather than a direct structure-to-killing correlation. Structural regulation first alters the coordination environment and electronic structure of active sites and then changes substrate binding, intermediate stabilization, electron transfer, and reaction energy barriers. These catalytic changes subsequently determine the type, amount, localization, and lifetime of bactericidal outputs, including ROS, HOX, metal ions, hydrolytic cleavage products, metabolic perturbation, or immunomodulatory signals. Only after interacting with infection-specific microenvironmental factors, such as pH, oxygen availability, hydrogen peroxide concentration, antioxidant levels, EPSs, and host immune components, do these catalytic outputs translate into measurable antimicrobial outcomes.

Importantly, structure–activity relationships in antimicrobial nanozymes should not be interpreted as universal linear rules. Their apparent effects are strongly influenced by nanozyme dose, dispersion state, bacterial species, assay medium, protein corona, biofilm maturity, and external stimulation conditions. Therefore, this review distinguishes generalizable design principles from context-dependent correlations whenever possible. In this section, we summarize the major structural parameters governing antimicrobial nanozyme performance and discuss how they can be rationally engineered to optimize catalytic activity, antimicrobial selectivity, antibiofilm efficacy, and translational potential.

### Chemical composition of nanozymes

**Fig. 3.**
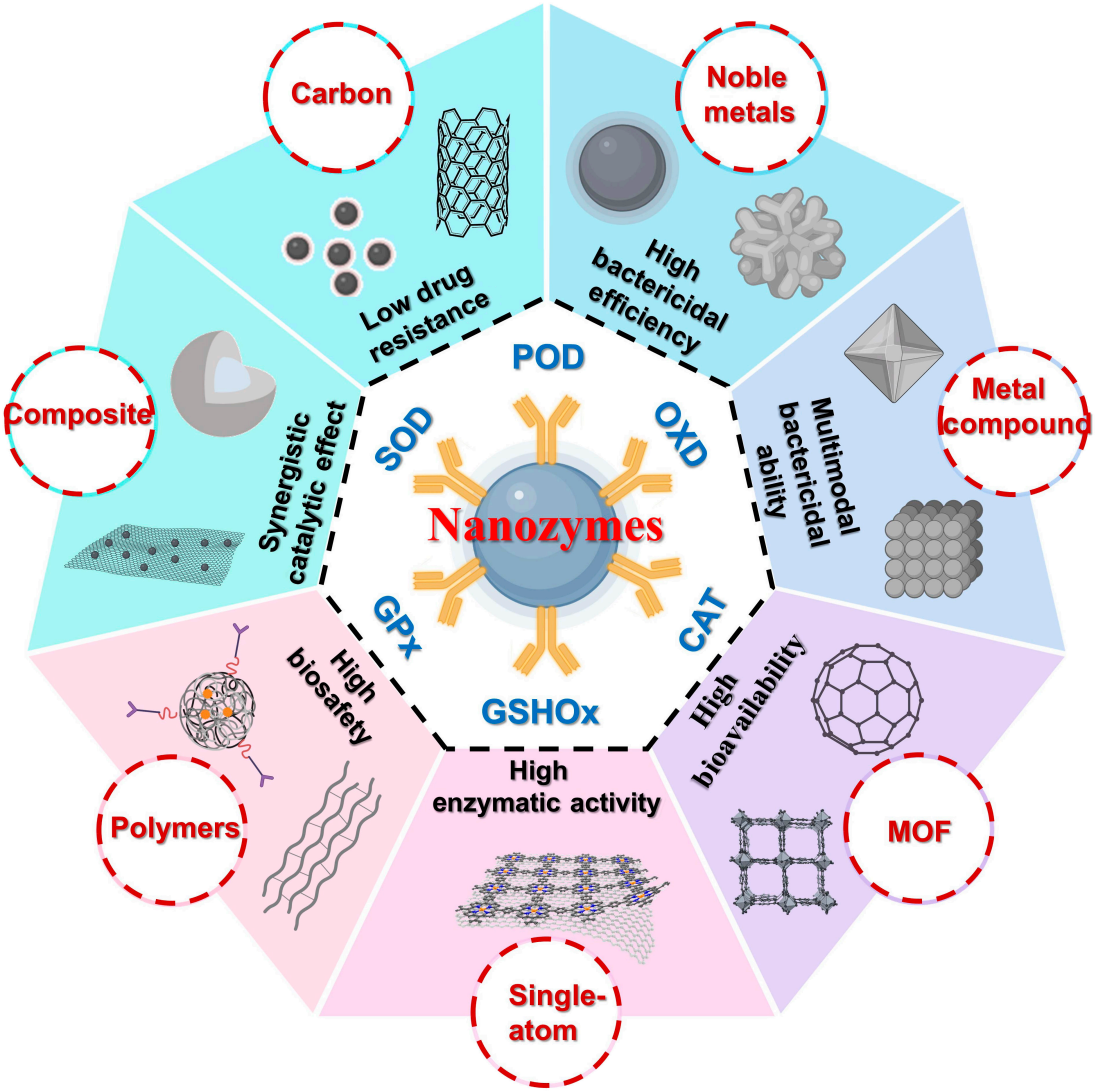
Classification of nanozymes by material category.

After academician Yan and her team [36] discovered the POD-mimetic activity of Fe_3_O_4_ nanoparticles (NPs) in 2007, the term “nanozymes” was formally proposed. Over the past decade, this research frontier has witnessed explosive progress, with more than 300 research groups globally working to advance related studies. A broad spectrum of nanozymes with enzyme-like activity have been reported, including carbon nanomaterials, noble metals, non-noble metals and their derivatives, MOFs, single-atom nanozymes, and organic–inorganic hybrid composites. The detailed material-based classification of nanozymes is shown in Fig. 3.

#### Carbon-based nanozymes

Carbon-based nanozymes include carbon dots (CDs) [37,38], carbon nanotubes (CNTs) [39], carbon nitride, fullerenes, graphdiyne (GDY) [24], carbon nanowires, graphene, GO [40], carbon aerogels, and others. They exhibit tunable catalytic performance, favorable recyclability, and outstanding structural stability. Moreover, their precisely controlled electronic and geometric architectures enable them to replicate the intricate catalytic active sites of native enzymes.

In particular, a “multi-atom co-doping” strategy can be adopted to synergistically boost the catalytic capacity of carbon nanozymes. The bactericidal capacity of carbon nanomaterials is mainly determined by raw material choices and elemental composition. While most pristine carbon samples display weak intrinsic antibacterial effects, their antimicrobial potency can be drastically elevated via surface engineering. For instance, surface charge and functional groups anchored on CD-based nanomaterials dominate binding interactions with bacterial cells and ultimately govern the materials’ bactericidal efficacy. Boukherroub and colleagues [41] reported ampicillin-functionalized CD nanozymes. Upon visible light irradiation, the nanozyme generated moderate levels of ROS and worked synergistically with ampicillin, which exerted prominent bactericidal effects against *E. coli*. While GDY possesses conductive, adsorptive, and catalytic characteristics, ZnO shows piezoelectric catalytic effects. By self-assembling a small amount of layered GDY nanosheets on the surface of synthesized ZnO nanorods (ZnONRs), ZnO@GDYNR POD-like nanozymes are fabricated [24]. Under ultrasound stimulation, abundant ZnO@GDYNR nanorods adhere to bacterial surfaces and trigger the formation of an internal built-in electric field. Such field effect facilitates the separation and transfer of charge carriers to the material surface, which accelerates H_2_O_2_ decomposition at catalytic active sites and produces abundant ROS to eradicate pathogenic bacteria. Benefiting from multi-modal bactericidal mechanisms, carbon-based nanozymes barely trigger bacterial drug resistance in contrast to conventional antibiotics. Accordingly, they have attracted extensive attention in nanozyme catalysis and possess great promise for various real-world applications [42].

#### Noble metal-based nanozymes

Noble metal nanozymes composed of silver, platinum, gold, palladium, ruthenium, and other metals have become an important research branch in antibacterial fields. Their antibacterial action relies on producing ROS via POD- and OXD-like activities, and they aggravate bacterial oxidative damage by consuming intracellular GSH. Furthermore, rational structural design endows noble metal nanozymes with multiple functions such as catalytic activity, photothermal/photodynamic performance, and sustained metal ion release, thus constructing a synergistic antibacterial system. For instance, silver itself exhibits inherent antibacterial properties. When combined with nanozyme catalytic activity, it delivers a synergistic bactericidal effect (1 + 1 > 2). Ag/Bi_2_MoO_6_ (Ag/BMO) nanozymes show NIR-II light-activated POD-like activity, photodynamic performance, and acid-responsive Ag^+^ release behavior [43]. Density functional theory (DFT) calculations revealed that Ag doping results in a reduced bandgap and improved charge separation in Bi_2_MoO_6_, thus enhancing the production of ROS. In vitro experiments demonstrated that the nanozymes exhibit a bactericidal rate of ~99.9% against methicillin-resistant *S. aureus* (MRSA) and favorable biocompatibility, with a cell viability of >80% and a hemolysis rate of <5% [44].

Although noble metal-based nanozymes exhibit relatively high bactericidal efficiency, the most critical challenge for alternatives to antibiotics is that their antibacterial mechanisms lack strict bacterial specificity, which is unable to efficiently kill bacteria without causing toxic damage to healthy mammalian cells and tissues. Furthermore, the high cost of noble metals and the difficulty in large-scale fabrication constitute another notable drawback of noble metal nanozymes.

#### Metal compound nanozymes

Another class of nanozyme materials are metal compounds, including metal oxides, metal sulfides, and metal phosphides. These nanozymes feature facile synthesis, tunable performance, good biocompatibility, and low cost. Metal oxide nanozymes such as cerium dioxide (CeO_2_), trimanganese tetroxide (Mn_3_O_4_), and vanadium pentoxide (V_2_O_5_) mimic antioxidant enzymes to scavenge excess ROS during inflammation, thus protecting cells against oxidative stress. In acidic environments with abundant H_2_O_2_, ferroferric oxide (Fe_3_O_4_) accelerates the generation of highly toxic ·OH and effectively destroys bacterial structures. Via oxygen vacancy regulation, CeO_2_ NPs successively exert SOD- and CAT-like activity. The redox cycle between Ce^3+^ and Ce^4+^ enables them to catalyze reactions with ·O_2_^−^ and H_2_O_2_. Additionally, some nanozymes achieve enhanced bactericidal activity via the synergistic release of metal ions or organic components. For iron sulfide-based nanozymes, sulfur species facilitate the iron redox cycle to sustain continuous ROS production; meanwhile, they participate in polysulfide formation, which further improves the overall bactericidal performance [45].

Metal compound nanozymes demonstrate distinct advantages in antibacterial applications through the synergistic combination of catalytic antibacterial activity and the intrinsic antimicrobial effects of metal ions or polysulfides. However, they are hindered by limitations including inadequate biocompatibility, limited stability under physiological conditions, and a tendency for catalytic activity to diminish over time.

#### Metal organic framework nanozymes

MOF nanozymes are constructed via the coordination assembly of metal ions and organic linkers, and they possess prominent merits including enzyme-mimetic activity, high porosity, and large specific surface area [32]. These materials feature tunable porosity and diverse morphologies, enabling effective regulation on the size of reactive molecules. Meanwhile, their high substrate selectivity further elevates catalytic activity. Additionally, the abundant surface and varied geometric structures of MOFs serve as an ideal platform for functional modification and activity optimization. Owing to the intrinsic porous structure and residual carboxyl groups, MOFs offer abundant metal ligand binding sites and thus facilitate the loading of Cu^2+^. Related studies have verified that Cu^2+^ released from MOF@Cu^2+^ can efficiently scavenge intracellular GSH in ROS-damaged biofilms. This effect greatly improves the photodynamic therapy (PDT) efficacy against *S. aureus* biofilm infections both in vitro and in animal models [46]. Fe_3_O_4_@MOF@Au NPs (FMANPs) incorporate ultrasmall Au nanoparticles (AuNPs) in situ into the pores of IRMOF-3, enhancing their POD-like activity through a cascade reaction [26]. Apart from excellent antibacterial biocompatibility, these NPs are capable of combating the Gram-negative (*E. coli*) and Gram-positive (*S. aureus*) bacteria. Their practical use is severely restricted by 2 key issues: unexpected degradation in physiological environments and inadequate solubility in certain solutions. Therefore, it is essential to further explore other MOFs and doping strategies to precisely regulate heteroatom composition, microstructural features, and spatial distribution, thus improving their real-world applicability.

MOFs are promising antibacterial candidates because of their good bioavailability. Their porous structures can encapsulate nanozymes and retain active sites, while rational modification of ligands or metal nodes enables targeted response to infection microenvironments. However, inherent limitations such as poor in vivo stability, cytotoxicity, complex synthesis, and high costs hinder clinical translation, and their widespread clinical use remains challenging.

#### Single-atom nanozymes

Single-atom nanozymes represent another class of materials similar to metal complex nanozymes. The concept of single-atom catalysts (SACs) was first coined by Zhang and colleagues [47] in 2011. Their work reported that isolated Pt atoms anchored on FeO*_x_* substrates showed high activity toward CO oxidation. For the synthesis of single-atom nanozymes, single metal atoms are immobilized on inorganic carriers such as MOFs, carbon materials, and metal oxides—typical preparation strategies include atomic layer deposition, high-temperature pyrolysis, and wet-chemical self-assembly. In these materials, isolated metal atoms serve as catalytic active sites. They are stabilized by forming unique coordination structures with surface atoms or functional groups of the supports, which effectively improves catalytic performance.

One remarkable superiority of single-atom nanozymes is their ability to address the inherent limitation of traditional NPs: the “inaccessibility of internal atoms to substrates”. Their theoretical active site utilization efficiency can reach 100%, which far outperforms that of conventional NPs (where only surface atoms typically participate in catalysis) or metal cluster nanozymes. Consequently, from a theoretical perspective, single-atom nanozymes show outstanding catalytic efficiency, rapid reaction kinetics, and unambiguous catalytic mechanisms. As described by Song et al. [48], an encapsulated pyrolysis strategy was employed to synthesize nanozymes consisting of single iron atoms anchored within nitrogen-doped amorphous carbon (SAF NCs). SAF NCs performed excellent POD-like activity in the presence of H_2_O_2_, producing toxic ·OH to achieve high-efficiency sterilization effect against *E. coli* and *S. aureus*. Similarly, a Mn single-atom nanozyme-functionalized 3D-printed bioceramic scaffold (Mn/HSAE@BCP scaffold) was developed [49]. The Mn/HSAE@BCP scaffold demonstrated clear CAT-, OXD-, and POD-like activities. Moreover, it was capable to generate sufficient ROS through a synergistic approach combining chemodynamic therapy (CDT) and sonodynamic therapy (SDT) to efficiently eliminate bacteria.

Single-atom nanozymes also have disadvantages such as inadequate substrate specificity, intricate composition, and limited active site density, and its long-term biodegradability and toxicity in vivo still require further exploration.

#### Polymeric nanozymes

Polymer nanozymes are macromolecules formed by covalent bonding of identical or similar monomer units. Regulating the functional groups of polymers and introducing extrinsic active sites can optimize the enzymatic activity, functionality, and biocompatibility of polymer–metal nanocomposites. Polymers widely applied in the antibacterial field mainly include but are not limited to chitosan, β-polylysine, peptides, and polydopamine [50]. Derived from organisms or natural products, these materials possess outstanding biocompatibility and non-immunogenicity. They rarely trigger severe inflammation or allergic responses in vivo, thus showing high biosafety.

To improve catalytic performance, polymers are usually combined with metals to fabricate composite nanozymes. For instance, AgNC@TP-I is a type of silver nanocluster green-synthesized with antimicrobial peptides as the reducing agent [51]. Owing to the positive surface charges provided by the modified peptides, the nanozymes can bind to *E. coli* via electrostatic interaction. In addition, hydrophobic interactions facilitate rapid cellular uptake of ultrasmall AgNC@TP-I, which disturbs bacterial metabolism and boosts intracellular ROS generation. Experimental results confirm that AgNC@TP-I presents excellent antibacterial activity. Meanwhile, the silver core protected by biomolecules endows the material with long-term antibacterial efficacy and low cytotoxicity. Accordingly, modulating biological interactions makes intracellular silver nanoclusters a promising candidate for the rational design of high-performance broad-spectrum antibacterial materials.

#### Composite nanozymes

Composite nanozymes are hybrid materials integrating 2 or more different components. Synergistic effects between components not only optimize the intrinsic performance of nanozymes but also endow them with new functions. These hybrids retain the inherent advantages of nanozymes, such as tunable catalytic activity, good structural stability, and facile synthesis. Meanwhile, they acquire extra merits including large specific surface area, as well as optical, electrical, and magnetic properties after hybridization. The synergistic effect among different components greatly boosts enzyme-mimetic activity and catalytic efficiency. By combining the strengths of diverse materials, composite nanozymes possess superior catalytic performance and broad application prospects. Typical composite nanozymes mainly include carbon-supported metal/metal oxide nanozymes, core–shell structured composites, polymer–metal oxide hybrids, alloy-based nanozymes, and MOF–NP composites. For example, iron-doped CDs (Fe-CDs) with an average diameter of 3 nm were prepared via a facile one-pot pyrolysis method [52]. Compared with pure CDs, Fe-CDs exhibited distinctly enhanced photo-boosted POD-like activity. Under NIR laser irradiation, Fe-CDs generated a photothermal effect. This effect eliminated bacteria efficiently without harming normal tissues and further improved catalytic efficiency. In vitro antibacterial tests proved that the Fe-CD-based platform achieved excellent bactericidal effects against *S. aureus* and *E. coli*. Notably, ultrasmall Fe-CDs also showed favorable biocompatibility. This dual-functional design offers a promising strategy to develop biocompatible, antibiotic-free nanomaterials for wound disinfection and healing. To address the key challenges in bone defect repair, including excessive osteoclast activation, insufficient osteoblast activity, and ROS-induced imbalance of inflammatory microenvironment, Li’s group designed a composite nanozyme named ezPatch. This nanozyme uses 2D Cu_3_(PO_4_)_2_ nanosheets as the core backbone. Its surface was first modified with gallic acid (GA), followed by further functionalization with tetracycline on the GA layer. Each component plays a unique role in the system: Cu_3_(PO_4_)_2_ nanosheets act as catalytic centers to release Cu ions and regulate bone metabolism; GA enhances catalytic activity and structural stability; tetracycline realizes bone-targeted anchoring; phosphate ions maintain crystal structure and mimic the mineral composition of natural bone [53]. Although this system was developed for bone-interface repair rather than antimicrobial therapy, it provides a useful example for refining nanozyme material categorization. It shows that composite nanozymes can be classified not only by chemical composition but also by dimensionality, interfacial organization, targeting modules, and therapeutic architecture. From a design perspective, such 2D patch-like nanozymes illustrate how catalytic centers, stabilizing ligands, targeting components, and tissue-mimetic structural elements can be integrated into a single platform. This classification logic is also relevant to antimicrobial nanozyme design, particularly for wound dressings, implant coatings, and localized infection interfaces where material adhesion, catalytic stability, and microenvironment regulation are simultaneously required.

By integrating the complementary strengths of different components, composite nanozymes exhibit versatile catalytic performance and tunable properties, as well as outstanding chemical and mechanical stability in complex environments. Since all single-component nanozymes have inherent limitations, composite nanozymes are expected to dominate future large-scale production.

### Physical structural parameters of nanozymes

Physical structural parameters provide the most direct handles for regulating nanozyme catalysis because they determine how active sites are exposed, how substrates approach the catalytic interface, and how electrons and reaction intermediates are transferred during antimicrobial reactions. Unlike chemical composition, which defines the elemental basis of catalytic activity, physical structure provides adjustable design handles that influence the spatial accessibility, local coordination, interfacial contact, mass transport, and biological fate of nanozymes. Therefore, parameters such as size, morphology, surface chemical state, surface modification, core–shell architecture, and defects should be analyzed not as isolated descriptors but as mechanistic variables that connect material design to catalytic output and antimicrobial performance.

**Fig. 4.**
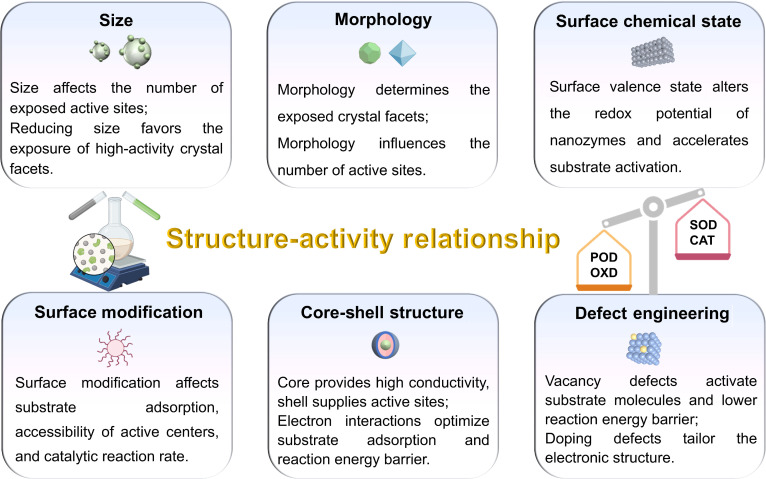
Structure–activity relationships of nanozymes: Influencing factors and mechanistic insights.

In antimicrobial applications, these structural parameters may influence at least 4 levels of performance: intrinsic enzyme-like activity, bacterial adhesion and penetration, biofilm matrix disruption, and host-related biosafety. For example, particle size affects active site exposure, aggregation, biofilm penetration, circulation, and clearance; morphology and exposed facets can regulate bacterial contact and reaction selectivity [54]; oxidation state and defects modulate redox cycling and reaction pathways; surface modification affects targeting, substrate affinity, and protein–corona interactions; and core–shell interfaces can facilitate electron transfer, substrate confinement, and cascade catalysis. These mechanistic relationships are summarized in Fig. 4 and Table 2, with emphasis on context-dependent trends rather than universal causal rules.

**Table 2.** Comparative structure–activity relationships of representative antimicrobial nanozymes

Factors	Nanozymes	Enzyme activities	Bactericidal mechanisms	Bacteria	Bactericidal performances	Ref.
Size	cPt/CNTs (10~70Pt/CNTs)	POD (30Pt/CNTs with the highest *K*_cat_)	·OH	*S. aureus*, *E. coli*	30 Pt/CNT nanozymes presented the highest antibacterial activity against *E. coli* (81%) and *S. aureus* (54%).	[141]
	Pt	POD + OXD (Pt_3.0nm_ > Pt_7.0nm_ > Pt_1.5nm_)	·OH and ·O_2_^−^	*S. aureus* Xen36, *E. coli Xen*14	The biofilm thickness order of *S. aureus* and *E. coli* is Pt_3.0nm_ > Pt_7.0nm_ > Pt_1.5nm_.	[142]
Morphology	Pd nanocrystals (cube{100}, octahedron{111})	POD + OXD ROS fluorescence intensity: Pd (cube) > Pd (octahedron)	·O_2_^−^, ·OH, sharp corners of octahedral Pd{111} nanoparticles, Pd cubes generated higher ROS than Pd octahedron	*S. aureus*, *E. coli*	Against *S. aureus*, cubic Pd ({100}) > octahedral Pd ({111}); against *E. coli*, octahedral Pd ({111}) > cubic Pd ({100})	[143]
	CeO_2_ cube(100), rod(110), octa(111)	POD-cube(100) + CAT + HPO-rod(110)	HOBr for octa(111), ·OH for cube(100), ·OH and HOBr for rod(110)	*E. coli*	CeO_2_ with octa(111) with the highest V_max_ for KBr displays the optimal antibacterial effect.	[58]
	PtCu (HDs, DFOs and octahedron)	POD (DFO > HD > octahedron) DFOs have a maximum V_max_ of 10.04 M·s−1 and the minimum *K*_m_ of 0.85 mM	·OH	*S. aureus*, *E. coli*	PtCu DFOs exhibit excellent antibacterial efficiency.	[144]
Surface chemical state	Cu^0^@C, CuO@C	POD (V_max_Cu^0^@HCS > V_max_CuO@HCS)	·OH for Cu^0^@C, Cu^2+^ for CuO@C	*S. aureus*, *E. coli*, *P. aeruginosa*	Cu^0^@C(·OH) and CuO@C(Cu^2+^) exhibit broad-spectrum antibacterial effect.	[62]
	CeO_2_ @ZIF-8 (higher Ce^3+^/Ce^4+^ ratio)	SOD + CAT (CeO_2_@ZIF > CeO_2_)	Zn^2+^, electrostatic interaction, and physical penetration	*E. coli*, MRSA *S. mutans*	Antibacterial efficacy of CeO_2_@ZIF-8 is 100%. CeO_2_ NPs were at about 97.20% for *E. coli*, 95.45% for MRSA, 95.04% for *S. mutans*.	[145]
Surface modification	MnFe_2_O_4_-NH_2_, MnFe_2_O_4_-COOH, MnFe_2_O_4_-PEG	POD (-NH_2_ ≈ -COOH > -PEG)	·OH, electrostatic interaction, and physical penetration	Gram-negative, Gram-positive bacteria, MRSA biofilm, *P. aeruginosa* biofilm	For MRSA T144 biofilm eradication efficiency, -NH_2_(65%) > -COOH (50%) > -PEG (35%). For *P. aeruginosa* biofilm eradication efficiency, -NH_2_ (85%) > -PEG (55%) > -COOH	[146]
Core–shell structure	Au@Co-Fe	POD	·OH	*P. Aeruginosa*, *B. cereus*, *E. coli*, and *S. aureus*	MIC: *E. coli* (3.25 mg/ml); *S. aureus* (0.91 mg/ml); *B. cereus* (0.91 mg/ml); *P. aeruginosa* (0.91 mg/ml)	[147]
	CeO_2_@C	HPO	HOBr	*S. aureus*, *E. coli*, *P. aeruginosa*	CeO_2_@C displayed excellent antibacterial effects against *S. aureus*, *E. coli*, *P. aeruginosa*.	[148]
Defect engineering	Ov-PMo12 (oxygen vacancy)	HPO	HOBr	*E. coli*	Ov-PMo12 can generate H_2_O_2_ via visible-light photocatalysis. HOBr can efficiently kills *S. aureus* and *E. coli*.	[149]
	Cu^+^-MoO_3–*x*_ (metal doping)	POD, V_max_/*K*_m_ = 2.32 (Cu^+^-MoO_3–*x*_ > MoO_3–*x*_)	·OH	*P. aeruginosa*	Damage ability of Cu^+^-MoO_3–*x*_ to *P. aeruginosa* is stronger than that of MoO_3–*x*_. The MBC of Cu^+^-MoO_3–*x*_ is 64 μg/ml at 2 h.	[150]

#### Size and surface area

Particle size and specific surface area are important structural parameters that influence the structure–activity relationship of nanozymes because they affect accessible active site density, substrate diffusion, aggregation behavior, biofilm penetration, and biological transport. Given that specific surface area is inversely proportional to particle size, reducing nanozyme size generally produces a larger surface area. A larger surface area exposes more surface atoms and active sites, facilitates substrate binding and interfacial interactions, and thus improves catalytic efficiency. Furthermore, particle size can regulate the exposure of crystal facets; smaller NPs tend to expose more highly active crystal facets. In addition, surface area strongly affects the local microenvironment around active sites such as surface charge and wettability, which further modulates substrate adsorption, intermediate stabilization, and product desorption. All the above factors jointly determine the catalytic activity and selectivity of nanozymes.

Particle size and surface area of nanozymes also play important roles in determining their dispersibility, aggregation behavior, and stability under biological conditions, all of which further govern their performance in real-world applications. Notably, the correlation between catalytic activity and particle size is not a simple linear relationship; instead, it follows a complex, nonmonotonic trend. In particular, an optimal particle size range exists where the enzyme-mimetic activity reaches its maximum. Activity declines when the particle size is either too small or too large relative to this optimal window. Although nanozymes with smaller dimensions feature higher specific surface areas and should theoretically provide more exposed active sites, extremely small particles tend to possess high surface energy, which induces severe aggregation and thus reduces the availability of active centers. Conversely, larger nanozymes offer fewer active sites owing to their lower specific surface area, yet they often display better colloidal stability and are less prone to aggregation. Furthermore, larger crystalline nanozymes may possess a more perfect crystal structure, which can improve the intrinsic catalytic efficiency of individual active sites. Several studies have demonstrated that the catalytic activity of nanozymes generally rises first and then declines with changing particle size, confirming that an optimal size exists.

For example, Yin’s group [55] synthesized gold nanozymes with different diameters and assessed their CAT-like activity by detecting ·OH generation in acidic environments. The results showed that CAT-like activity increased as Au particle size grew from 10 to 50 nm, yet dropped slowly when the size further increased to 100 nm. In another related work, Yu and colleagues [56] prepared iron oxide nanozymes of various sizes and observed the same trend for POD-like activity. This phenomenon is mainly ascribed to the higher catalytic activity of Fe^2+^ compared with Fe^3+^. For small particles, Fe^2+^ is more prone to oxidation, and electronic structure becomes the primary factor governing POD-like activity. In contrast, specific surface area dominates for larger particles. Accordingly, iron oxide nanozymes show nonmonotonic activity versus particle size, with the maximum activity achieved at a moderate size.

Nevertheless, the rise-then-fall trend of catalytic activity with increasing particle size is not universal among nanozymes. Although widely reported in size-dependent studies, this rule does not apply to all cases, as the actual size–activity relationship is controlled by multiple factors. First, inherent material properties cause different nanozymes (metal oxides, noble metals, and carbon-based nanomaterials) to respond differently to size variation. Second, catalytic performance is tightly linked to substrate interactions, so optimal particle sizes vary for different reactions. Surface functionalization can also alter the exposure of active sites and substrate-binding affinity. Furthermore, external conditions such as pH, temperature, and ionic strength indirectly regulate the correlation between particle size and catalytic activity.

From a design perspective, particle size should not be optimized solely to maximize surface area. Instead, it should be selected according to the intended antimicrobial scenario. Smaller nanozymes may favor biofilm penetration, bacterial contact, and renal clearance, whereas larger or structured nanozymes may offer improved colloidal stability, prolonged local retention, and reduced nonspecific cellular uptake. Therefore, the optimal size window is context-dependent and should be evaluated together with catalytic pathway, infection depth, delivery route, and biosafety requirements.

#### Morphology

Morphology is a vital structural factor that strongly affects the catalytic activity of nanozymes. It determines surface characteristics such as specific surface area, active site exposure, surface electronic states, and substrate-binding affinity. Many studies have confirmed the close relationship between nanozyme morphology and enzyme-mimetic performance. For example, Mugesh and colleagues [57] synthesized orthorhombic V_2_O_5_ nanozymes with diverse morphologies, including nanowires (VNw), nanospheres (VSp), nanoflowers (VNf), and nanosheets (VSh). The results proved that these V_2_O_5_ nanostructures possessed marked GPx-like activity. Exposed crystal facets also play a decisive role in catalysis, because surface reactivity and redox properties rely heavily on surface atomic arrangement. Morphological differences change surface electron density and band structure, and further regulate overall catalytic performance. Peng’s group [58] prepared 3 types of CeO_2_ nanostructures with octahedral, rod-like, and cubic shapes. These samples exhibited distinct enzyme-mimetic activities. The coordination and electronic structures of surface Ce sites vary with morphology, which changes the activation pathway of H_2_O_2_ and tunes catalytic behavior. Among them, cubic CeO_2_ shows nearly 100% selectivity toward POD- and CAT-like activities, while octahedral CeO_2_ presents high selectivity only for CAT-like activity. Such differences originate from the varied coordination environments and electronic states of surface Ce sites in different CeO_2_ morphologies, which alter H_2_O_2_ activation and eventually define the type of enzyme-mimetic activity. These findings demonstrate that morphology regulates nanozyme catalysis by reshaping exposed facets, surface atomic arrangements, electron-density distribution, and bacterial-contact interfaces. For antimicrobial design, morphology should therefore be considered not only as a factor affecting catalytic activity but also as a determinant of bacterial adhesion, membrane contact, biofilm penetration, and catalytic selectivity.

#### Surface chemical state

Besides particle size and morphology, which greatly affect nanozyme catalysis, tuning surface chemical states is another important strategy to regulate catalytic performance. Surface chemical properties are critical to the structure–activity relationship of nanozymes. In most cases, catalytic performance is governed by active sites with specific surface chemical characteristics, which directly determine site activity. These surface states adjust interfacial interactions between nanozymes and substrates, such as electrostatic force, hydrogen bonding, and coordination interactions, thus mediating the whole catalytic process. Moreover, rational regulation of surface chemical states allows targeted optimization of nanozyme activity, selectivity, and stability.

Studies have indicated that the surface oxidation state of CeO_2_ plays a vital role in regulating its enzyme-mimetic properties. Pirmohamed and colleagues [59] illustrated that a decreasing Ce^3+^/Ce^4+^ redox ratio is associated with an increased rate of H_2_O_2_ decomposition. On the contrary, a lower Ce^3+^/Ce^4+^ ratio leads to a decline in SOD-like activity [60]. This phenomenon is not exclusive to CeO_2_ nanozymes. Wei and colleagues [61] reported that porous LaNiO_3_ nanocubes, which contain Ni^3+^, possess POD-like activity that is roughly 58 times higher than that of NiO (featuring Ni^2+^) and 22 times higher than that of Ni NPs (with Ni^0^). This finding underscores the strong dependence of the POD-like activity of nickel-based nanozymes on the nickel’s oxidation state. Likewise, the enzymatic activity of copper-based nanozymes is affected by the oxidation state of copper. Gao and colleagues [62] fabricated 2 types of copper/carbon nanozymes, namely, CuO-HCSs and Cu-HCSs. CuO-HCSs show relatively low POD-like activity and achieve bactericidal effects by releasing copper ions. In contrast, Cu-HCSs exhibit high POD activity, which enables efficient sterilization through the decomposition of H_2_O_2_ to generate ·OH. Collectively, these examples indicate that surface chemical state is a mechanistic determinant of redox cycling, electron transfer efficiency, and catalytic direction. For antimicrobial nanozymes, controlling oxidation state is particularly important because it can switch a material between ROS-generating, ROS-scavenging, ion-releasing, and inflammation-regulating functions. Thus, valence-state regulation should be evaluated together with antimicrobial output and host-tissue safety rather than treated as an isolated catalytic parameter.

#### Surface modification

Surface modification represents an effective strategy to modulate the catalytic activities of nanozymes. Various moieties including amino acids, ligands, aptamers, and cyclodextrins can be introduced onto the nanozyme surface to construct hybrid nanozyme systems, thereby optimizing their catalytic performance. Surface modifiers can tailor the surface charge, hydrophilicity, and hydrophobicity of nanozymes, which further influence the adsorption and binding affinity between substrates and nanozymes. Additionally, they can optimize the electronic structure of active sites, regulate steric hindrance, improve the accessibility of catalytic centers, and facilitate electron transfer, ultimately altering the catalytic rate of nanozymes and enabling efficient regulation of their enzymatic activities. For instance, Fan et al. [63] employed Fe_3_O_4_ nanozymes as a model platform to address the limitations of low intrinsic POD-like activity and weak substrate affinity. Drawing on the active site architecture of natural PODs, they immobilized histidine onto the surface of Fe_3_O_4_ NPs to mimic the microenvironment of native enzymes. The results demonstrated that histidine modification enhanced the binding affinity (*K*_m_) of Fe_3_O_4_ nanozymes toward H_2_O_2_ by more than 10-fold, and improved the catalytic efficiency (*K*_cat_/*K*_m_) by up to 20-fold, leading to a remarkable enhancement in POD-like catalytic performance. This enhancement is attributed to the improved substrate binding, electron transfer, and local microenvironment induced by amino acid modification. Subsequently, Fan et al. [15] systematically investigated the effects of different surface ligands: poly(sodium 4-styrenesulfonate) (PSS), poly(vinyl alcohol) (PVA), poly(vinyl pyrrolidone) (PVP), and poly(acrylic acid) (PAA) on the POD-like activity of Ru nanozymes. Ru nanozymes modified with PSS exhibited the highest catalytic activity, with a specific activity of 2,820 U mg^−1^, which was twice that of natural HRP. DFT calculations revealed that PSS regulated the electronic structure of Ru by withdrawing electrons from its surface, reducing the binding affinity between Ru and the reactive intermediate •OH, and thus greatly accelerating the catalytic cycle.

It remains a major challenge for nanozymes to combine high catalytic efficiency with favorable substrate selectivity during enzyme biomimicry. Willner’s team [64] pioneered aptamer-functionalized nanozymes (aptananozymes). With Cu^2+^-functionalized carbon dots (Cu^2+^-C-dots) serving as catalytic cores, they covalently immobilized substrate-specific aptamers. The resulting nanozymes exhibit enhanced catalytic activity and can oxidatively degrade dopamine. This strategy sheds light on the selective removal of targeted environmental pollutants.

These examples show that surface modification provides a bridge between catalytic regulation and biological specificity. Ligands, amino acids, aptamers, polymers, and biomimetic coatings can tune substrate affinity, intermediate binding, electron transfer, bacterial adhesion, protein–corona formation, and immune cell interactions. Therefore, surface engineering is especially important for antimicrobial nanozymes because it determines whether catalytic activity generated in model reactions can be translated into selective activity at bacterial membranes, biofilm matrices, or infected tissues.

#### Core–shell structure

In general, material structure dominates its intrinsic properties. Core–shell structure is a classic composite architecture widely adopted to modulate the enzyme-mimetic activity of nanozymes, which consists of an inner core and one or multiple outer shell layers coating the inner core. The core and shells are linked through interfacial bonding to form an integrated structure. Typically, core and shell components are both constructed from high electronic-conductivity materials, including metals and metal oxides. The continuous core–shell interface accelerates electron transfer—a key rate-limiting step in catalytic reactions, and further boosts catalytic reactions. Moreover, the outer shell optimizes nanozyme surface characteristics (surface charge, functional groups, hydrophilicity, and hydrophobicity), which strengthens substrate adsorption affinity. Such modification facilitates substrate enrichment around active sites and ultimately elevates the overall catalytic efficiency.

Nanozymes with a core–shell structure hold marked advantages in enhancing enzymatic activity, the advantage stemming from the deliberate design of core and shell compositions, interfacial interactions, and surface properties. The core and shell materials typically possess complementary physicochemical characteristics, and their interfacial interactions—such as electron transfer and synergistic adsorption—generate a “1 + 1 > 2” catalytic effect that surpasses the performance of individual components. For instance, Karyakin and colleagues [65] developed core–shell structured PB-NiHCF nanozymes, where the Prussian Blue (PB) core and NiHCF shell exhibit complementary properties: The PB core provides excellent electron conductivity, while the NiHCF shell enhances substrate adsorption capacity, resulting in a catalytic rate constant far superior to that of single-component nanozymes. Similarly, Kong’s group [66] reported a MnOx@EGaIn core–shell nanozyme, in which the EGaIn core ensures efficient electron transfer, and the MnOx shell optimizes substrate binding, enabling the nanozyme to exhibit both high POD-like activity and good stability. Another example is the Au@Pt core–shell nanozymes designed by Wang and colleagues [67], where the Au core provides stable structural support, and the Pt shell enhances substrate adsorption and electron transfer efficiency. This core–shell design effectively prevents Pt NP aggregation, drastically improving the accessibility of active sites and thus boosting the POD-like catalytic activity.

Notably, the core–shell structure not only maximizes the specific surface area and active site exposure but also protects the core material from harsh reaction environments (extreme pH, high temperature), ensuring long-term catalytic stability. This structural design offers a feasible strategy for regulating the enzymatic activity of nanozymes, highlighting the importance of structural engineering in optimizing catalytic performance.

From a mechanistic viewpoint, core–shell engineering is particularly valuable when a single component cannot simultaneously provide high catalytic activity, stability, targeting, and biosafety. The core can serve as an electron transfer reservoir, structural stabilizer, or photothermal component, while the shell can regulate substrate access, bacterial interactions, ion release, and biocompatibility. Therefore, core–shell design should be evaluated as an interfacial catalytic strategy rather than merely a composite structure.

#### Defect engineering

Defect engineering techniques have been widely used to enhance the catalytic activity of nanozymes. H_2_O_2_ oxidative etching, chemical etching, formamide solvothermal defect induction, and vacancy generation via reductant reduction are common approaches for introducing structural defects into nanozymes [68]. Defect engineering describes the intentional introduction of defects into materials to precisely tailor the active sites of nanozymes and attain target catalytic performance. Such defects are generally categorized as point, line, planar, and bulk types, differentiated by their geometric structures and formation mechanisms.

Vacancy defects refer to missing atoms or ions that should occupy fixed lattice sites in crystals, leaving empty lattice positions. Oxygen vacancies are the most common type, alongside sulfur vacancies, metal vacancies, and other cation/anion vacancies. Such vacancies modulate nanozyme enzyme-mimetic activity via 3 core pathways: reshaping the coordination environment of active sites, tuning electronic structures, and strengthening substrate-active site interactions. For instance, Yu et al. [69] synthesized oxygen vacancy-rich CoFe quantum dot (CoFe QD) nanozymes with distinctly improved POD-like activity. DFT calculations confirmed that oxygen vacancies adjust electronic configurations, lower reaction energy barriers, and facilitate the formation of ·OH. Using these vacancy-engineered CoFe QDs, the group constructed a colorimetric sensor for rapid on-site detection of small molecules including glucose and GSH.

Doping defects constitute a class of point defects generated by the incorporation of heteroatoms into the crystalline lattice of a host material. These foreign atoms can either substitute for the original lattice ions or occupy interstitial sites, thereby perturbing the inherent periodicity of the crystal structure. The range of dopant atoms is broad, encompassing various metal ions as well as nonmetal species such as Cl, S, and N. At the atomic level, doping defects typically modulate enzyme activity by restructuring the local coordination environment of active sites, tailoring the electronic band structure, and engineering the surface microenvironment. A notable example is the work by Lu et al. [70], who synthesized Fe-doped MoO*_v_* nanozymes (referred to as Fe-MoO*_v_*). The introduction of Fe ions induced distinct structural reconstruction in the MoO*_v_* lattice, which synergistically improved both the substrate-binding affinity and intrinsic catalytic performance of the nanozyme. Furthermore, the Fe-MoO*_v_* nanozymes were capable of mimicking the cascade reactions of natural enzymes. By inducing a profound disruption of redox and metabolic homeostasis within tumor microenvironments, the nanozyme exhibited markedly enhanced antitumor efficacy [69].

Edge dislocations are typical fundamental linear defects in crystalline materials. Their catalytic mechanism relies on reconstructed coordination environments of active sites, tuned electronic structures, and accelerated substrate mass transport driven by local lattice distortion and stress fields. For example, Fan and colleagues [71] fabricated defective Fe-N_4_ single-atom nanozymes via edge-site engineering. Edge sites in such structures offer extra anchoring sites for Fe single atoms, which modulate the electronic configuration of Fe-N_4_ moieties and accelerate catalytic decomposition of H_2_O_2_. These nanozymes exhibit promising capacity to relieve oxidative stress linked to retinal vasculopathies.

Grain boundaries, a typical type of planar defect, are atomically disordered interfaces formed between crystallites with different orientations in nanozymes, such as metal/metal oxide NPs and 2D nanosheets. These interfaces intrinsically create dense catalytic active sites including coordinatively unsaturated metal sites and variable-valence sites while facilitating fast electron transport and thereby elevating enzyme-mimetic activity. Grain boundaries improve catalytic performance mainly due to abundant unsaturated metal sites, mixed-valence metal pairs, and unobstructed electron transfer channels. For instance, Zhu and colleagues [72] synthesized grain boundary-rich ceria metallene nanozymes, whose phosphatase-like activity was 49 times higher than that of defect-free CeO_2_. Such dramatic activity improvement originates from the structural distortions at grain boundaries, which act as lattice cracks and edges enriched with coordinatively unsaturated atoms. These distorted zones contain large amounts of mixed-valence Ce^4+^/Ce^3+^ pairs, which mimic the active sites of natural phosphatases. Meanwhile, lattice strain and boundary defects tune the material’s electronic structure and accelerate electron shuttling between Ce^4+^ and Ce^3+^. This greatly speeds up the whole catalytic cycle, particularly the hydrolysis of phosphodiester bonds.

Apart from the structural defects mentioned above, voids and heterostructure defects also play vital roles in tuning nanozyme performance. At the atomic and electronic levels, defect engineering modulates nanozyme structure and catalytic behavior by changing coordination unsaturation, local charge distribution, adsorption energy, electron transfer pathways, and reaction energy barriers. For antimicrobial applications, such regulation can increase ROS or HOX generation, promote hydrolytic cleavage of biofilm components, or alter redox-homeostasis modulation. However, excessive or poorly controlled defects may also reduce structural stability, accelerate metal ion leakage, or increase off-target toxicity. Therefore, defect engineering should be considered a strategy for balancing catalytic potency, selectivity, stability, and safety rather than simply maximizing the number of active sites.

### Unresolved mechanistic questions in structure–activity-guided antimicrobial nanozyme design

Despite rapid progress in structure–activity-guided nanozyme design, several mechanistic questions remain unresolved. First, catalytic activity measured with model substrates does not necessarily represent catalytic behavior in infection-relevant environments, where proteins, salts, serum components, EPSs, and bacterial metabolites may passivate active sites or alter substrate accessibility. Second, the true active sites of many antimicrobial nanozymes under biological conditions remain difficult to identify, because oxidation state, defect density, ion release, surface ligands, and protein corona can dynamically change during treatment. Third, the relative contributions of ROS generation, metal ion release, membrane disruption, metabolic interference, hydrolysis, and immune modulation are often difficult to deconvolute in complex infection models. Fourth, most reported structure–activity relationships are derived from independent studies using different materials, bacterial species, culture conditions, and evaluation methods, which limit direct comparison and causal interpretation.

Addressing these questions requires more rigorous mechanistic tools and standardized models. Isotope tracing, quantitative ROS or HOX probes, electron paramagnetic resonance, in situ imaging, infection models, and kinetic modeling may help clarify active site evolution and reaction pathways under biologically relevant conditions. Nanozyme-assisted analytical platforms, including microfluidic biosensing systems, also provide useful methodological perspectives for real-time monitoring and performance benchmarking. Such approaches will be essential for transforming structure–activity relationships from descriptive correlations into predictive design principles.

To provide a more critical comparison, Table 2 summarizes how different structural parameters contribute to distinct levels of antimicrobial nanozyme performance. In general, oxidation state, defect density, and interfacial structures more directly affect intrinsic redox cycling and catalytic output, whereas particle size, morphology, and surface modification strongly influence bacterial adhesion, biofilm penetration, biodistribution, and biosafety. Nevertheless, these factors do not act independently. Their relative importance depends on material composition, bacterial species, infection microenvironment, external stimulation conditions, and evaluation methods. Therefore, the comparison in Table 2 should be interpreted as a context-dependent design guide rather than an absolute ranking of structural parameters.

## Synergistic Bactericidal Modes of Nanozymes

**Fig. 5.**
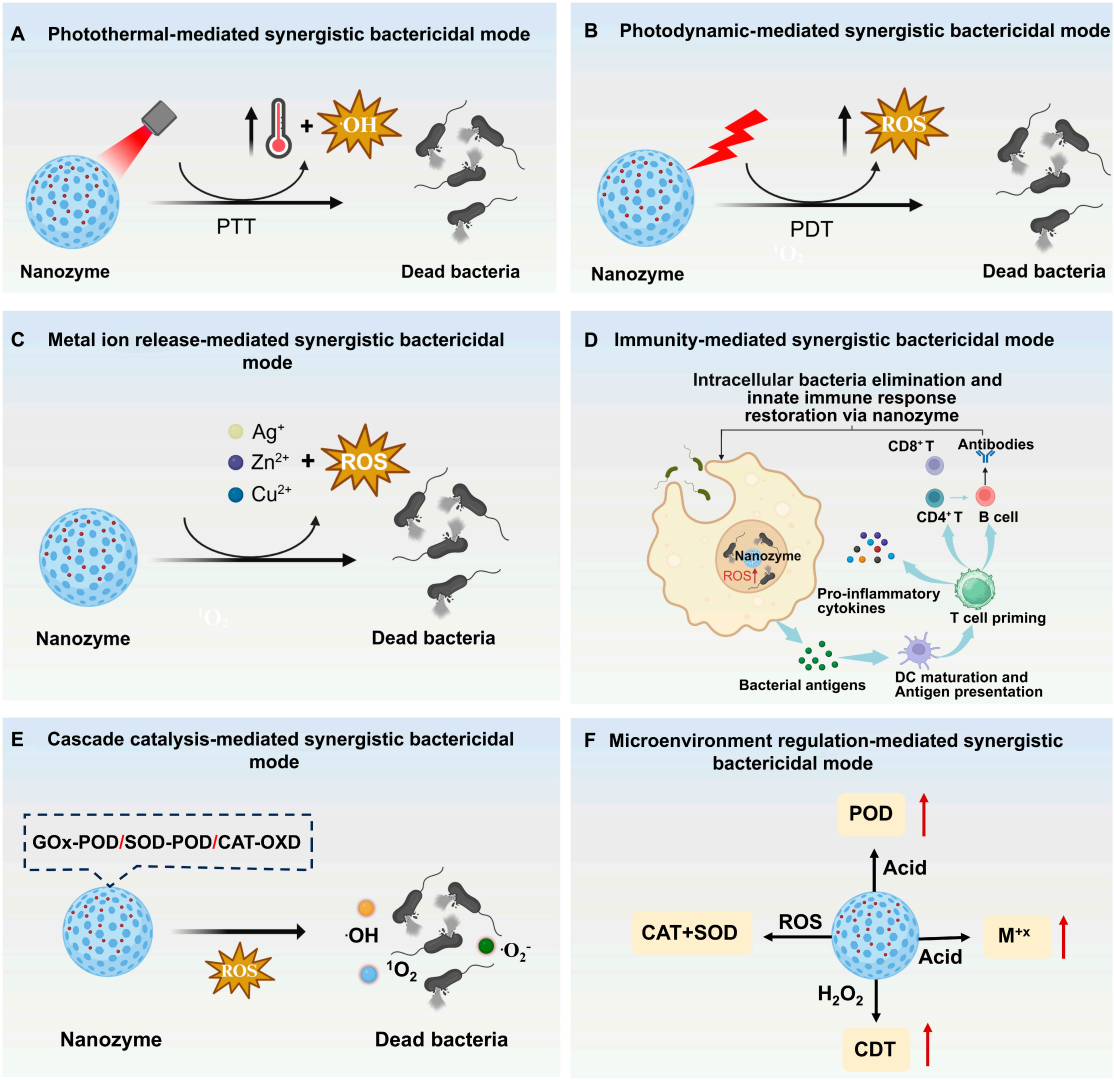
Schematic illustration of representative antibacterial mechanisms of nanozymes (A-F).

By introducing external conditions (light, heat, ultrasound, magnetic fields, electric fields, etc.) into the catalytic reaction system of nanozymes, researchers can employ a controllable and versatile strategy to enhance enzyme-like activity without affecting the nanozymes’ intrinsic properties. In nanozyme-mediated antibacterial therapy, external stimuli-driven energy conversion can be integrated with nanozyme catalytic reactions. This integration enables the continuous conversion of external energy and chemical compounds (such as water and oxygen) in the bacterial microenvironment into bactericidal agents like ROS and heat, effectively eliminating bacteria. Notably, these bactericidal agents are able to specifically target the infected site through irradiation without being blocked by the defense system. However, the regulation of nanozyme activity continues to present difficulties, given that the oxidation process can be activated spontaneously in aqueous environments, causing harmful side effects to healthy tissues. Therefore, in recent years, synergistic antibacterial approaches combining nanozymes with immune responses, multi-enzyme cascades, and microenvironment-responsive mechanisms have also attracted extensive research attention. The common antibacterial modes of nanozymes are summarized in Fig. 5. For example, nanozymes can raise local temperatures under photothermal effects to achieve enhanced bactericidal efficacy (Fig. 5A). Nanozymes boost ROS production via photodynamic therapy, thereby strengthening their bacterial killing capacity (Fig. 5B). Metal ions released from nanozymes synergized with ROS can realize highly efficient elimination of bacteria (Fig. 5C). Nanozymes execute direct catalytic bactericidal effects and simultaneously activate host innate immunity to achieve synergistic anti-infection therapy (Fig. 5D). Cascade catalytic reactions mediated by nanozymes continuously amplify intracellular ROS, disrupt bacterial membrane integrity and metabolic homeostasis, and ultimately trigger efficient bacterial death (Fig. 5E). In acidic infectious microenvironments, the nanozyme displays elevated POD-like activity, accelerated metal ion release, and strengthened H₂O₂-mediated CDT; its intrinsic CAT/SOD-like activities regulate ROS homeostasis to realize multi-pathway synergistic sterilization (Fig. 5F).

### Photothermal synergy-mediated bactericidal mode

Photothermal therapy (PTT) is a therapeutic approach featuring remote controllability, low drug resistance, and minimal invasiveness. Nanozymes exert antibacterial effects by generating ROS, which can lead to lipid peroxidation and protein denaturation in bacterial cell membranes, ultimately destroying bacterial cells [73]. PTT irradiates tissues with specific-wavelength light to elevate local temperature and initiate photochemical reactions. Such reactions inflict thermal and photodynamic damage on bacteria to strengthen antibacterial outcomes. In addition, following the Arrhenius law, the catalytic activity of nanozyme materials rises as temperature increases. Nanozymes with high photothermal conversion efficiency synergistically boost CDT by accelerating Fenton reactions under laser irradiation, which facilitates more ·OH production [74]. Combining nanozymes with PTT can generate higher temperatures under light irradiation, accelerating ROS production and the bactericidal process. This synergistic interaction can markedly improve the efficiency and effectiveness of antibacterial activity, particularly against drug-resistant bacteria. Therefore, PTT offers a potential alternative to address the drug resistance issues encountered in conventional antibiotic therapies. Moreover, owing to its spatiotemporal controllability, moderate tissue penetrability, high antibacterial efficacy, broad-spectrum activity, and low risk of drug resistance, this approach has emerged as a promising strategy for treating cutaneous and ocular infections [49]. Nevertheless, obstacles such as restricted enzyme-like catalytic capabilities, suboptimal catalytic conditions, poor interfacial interactions between nanozymes and bacteria, unsafe laser irradiation settings, and ineffective management of wound fluid impede both their antimicrobial performance and the speed of wound repair. These challenges further highlight promising avenues for future investigations. Studies have shown that ICG@hMnOx exhibits excellent photothermal performance, capable of disrupting dense biofilms and enhancing the efficiency of POD enzyme activity for sterilization [75]. Other PTT researches are summarized in Table 3.

**Table 3.** Photothermal synergy-mediated nanozymes for antibacterial applications

Strategies	Nanozyme	Enzyme-like activities	Antibacterial mechanisms	Bacteria	Bactericidal performances	Ref.
Nanozyme + NIR	PHMoD	CAT	ROS + PTT	*E. coli*, *S. aureus*	For *S. aureus*, survival rate is 0.24%, for *E. coli* (0.58%)	[151]
	PNMn	POD + OXD + CAT	ROS + PTT (η = 31.8%)	*E. coli*, *S. aureus*	Bactericidal efficacy is nearly 100%	[152]
	IR/P@Ce	POD (K_m_H_2_O_2_ = 6.67 mM)	·OH + PTT + PDT (η = 37.4%)	*E. coli*, *S. aureus*	99% bacterial reduction within 5 min	[153]
	IC-DAN	POD (*K*_cat_H_2_O_2_ = 4.92 S^−1^)	·OH + PTT	MRSA, MREC	Bactericidal efficacy is 100%	[154]
	Cu SASs/NPC	CAT + GSH-Px	ROS + PTT (η = 82.78%)	*E. coli*, MRSA	Bactericidal efficacy is 100%	[155]
	GDY-Fe@HA-DA	POD	·OH + PTT	*E. coli*, *S. aureus*, *P. gingivalis*	More than 95% antibacterial effect against bacteria	[156]
	Fe-CDs	POD (K_m_H_2_O_2_ = 97.64 mM)	·OH + PTT (η = 35.11%)	*E. coli*, *S. aureus*	Bactericidal rate was 99.68% for *S. aureus* and 99.85% for *E. coli*	[52]
	GZNC	POD (K_m_H_2_O_2_ = 2.115 mM)	·OH + PTT + Botanicals	Intracellular MRSA	Bactericidal rate exceeds 90%	[157]
	Ti_3_C_2_Tx-Se NPs	CAT + SOD	ROS + PTT (η = 61.98%)	*E. coli*, *S. aureus*	Antibacterial rates of 99.28% against *S. aureus* and 99.85% against *E. coli*	[158]
Nanozyme + NIR + NO therapy	PBzymes + SPBzyme	CAT + POD + SOD	ROS control + PTT + NO	*Fusobacterium nucleatum*, *P. gingivalis*	SPBzyme + NIR exerts a potent effect on *P. gingivalis* biofilms	[159]

### Photodynamic synergy-mediated bactericidal mode

Antibacterial PDT offers a non-antibiotic alternative by utilizing light irradiation to activate photosensitizers (PSs), which generate ROS that cause irreversible oxidative damage to bacteria, enabling their selective eradication. By effectively targeting bacterial pathogens without inducing antibiotic resistance, PDT has positioned itself as one of the strongest antimicrobial strategies currently in use. For efficient PDT, PSs are indispensable and can be classified into type I and type II according to their ROS generation mechanisms. Most existing PSs operate via the type II mechanism, which generates ^1^O_2_ through energy transfer from the excited triplet-state PS (3PS) to surrounding molecular oxygen. In contrast, type I PSs undergo photoinduced electron transfer (PeT) with nearby components (substrates, water, or oxygen), yielding ·OH or ·O_2_^−^. Notably, compared to the highly oxygen-dependent type II PSs, type I PSs demonstrate minimal or even no oxygen dependence [76]. Compared to traditional antibacterial drug therapies, PDT offers advantages such as low toxicity, broad-spectrum activity, minimal invasiveness, and a favorable safety profile. However, the chemical properties of PSs limit its accumulation and ability to penetrate biological tissues and cells, resulting in low efficiency of ROS generation. Additionally, PSs suffer from poor targeting of bacteria, which greatly hinders the implementation of PDT. Consequently, new strategies must be developed to boost PDT effectiveness. Nanozymes, with their ability to increase ROS generation and improve targeting, can address these limitations, thereby enabling more effective bacterial eradication. Studies indicate that Ce-loaded Prussian blue nanoparticle (CPBNP) nanozymes are capable of chemically conjugating with Ce6 while simultaneously demonstrating outstanding nanozyme activity and oxygen production capabilities. These properties can serve to relieve hypoxia in the bacterial environment, enhance Ce6-mediated PDT effectiveness against MRSA, and facilitate diabetic wound repair [77]. Studies on the PDT are listed in Table 4. In conclusion, the integration of nanozymes with PDT presents a new strategy for tackling bacterial infections, demonstrating promising potential, particularly in combating drug-resistant bacteria and complex infection microenvironments [78,79].

**Table 4.** Photodynamic synergy-mediated nanozymes for antibacterial applications

Strategies	Nanozyme	Enzyme-like activities	Antibacterial mechanisms	Bactericidal performances	Ref.
Nanozyme + PDT	CeCyan-Cu5.4O	CAT + SOD + GPx	PDT	Bactericidal rates are 100% against *P. gingivalis*, *F. nucleatum*, and *P. acnes*	[160]
	Cu-TCPP@MnO_2_	Fenton + CAT	PDT + CDT + PTT (η = 39.93%)	For *E. coli*, 95.9% inhibition rate, 99.9% for *S. aureus*; nearly 80% biofilm removal rate	[161]
	S-MM@CeO_2_-TCPP	POD (K_m_H_2_O_2_ = 3.85 mM)	Toll-like receptor anchoring + ·OH + PDT	Suppresses bacterial growth partially agains*t E. coli*, *S. aureus*	[162]
	Fe-Ce6 NPs	POD	·OH + PDT	Antibacterial rate exceeding 95% against both *S. aureus* and *E. coli*	[163]
	Co_7_Fe_3_/ZnO@C	POD (*K*_m_ = 5.35 × 10^−2^ mM) + SOD (*K*_m_ = 2.07 mM) + CAT (*K*_m_ = 2.07 mM)	·OH + PDT + PTT (η = 29.1%)	Excellent antibacterial performance *E. coli*, *S. aureus*	[164]
	HEPP	POD + OXD + GOx + CAT	ROS + PDT + PTT (η = 30.1%) + ES + macrophage polarization	Absorbance of bacteria at 600 nm decreased by ≈93% against MRSA, *P. aeruginosa*, PDR-PA	[165]
	UCNP@MOF	CAT	PDT + CDT + release of Cu^2+^ + Cu^2+^/Cu^+^ cycle	The inhibition rates reached 99.5% and 99.6% against *E. coli*, *S. aureus*; effectively inhibit biofilm formation	[166]
	M-GQDs@Ru-NO	POD	PDT + CDT + PTT + NO + ·OH	Remarkable antibacterial efficacy against MRSA	[167]
	CN-MnO_2_/PLLA	POD + CAT + GPx	ROS + PDT	99% and 98.7% elimination of *S. aureus* and *P. aeruginosa*	[168]
	DHTPY-Cu@ZOL	CAT	PDT + regulate ROS production	>90% antibacterial capability against MRSA	[169]

### Metal ion release-mediated synergistic bactericidal mode

Metal ion release-mediated bactericidal mode refers to the utilization of certain metal materials to continuously and controllably release bioactive metal ions in specific environments (such as bodily fluids and moist surfaces). These ions interact with bacteria through various physicochemical pathways, disrupting their critical physiological functions and ultimately leading to bacterial death. Ag^+^, Cu^2+^, and Zn^2+^ are effective species to kill bacteria through direct disruption of the bacterial membrane and proteins. This mode does not rely on traditional antibiotics, making it an effective strategy to combat bacterial resistance. For example, Jiang et al. [80] reported a composite hydrogel (OBG@CG). Cu^2+^ released from the Cu_2-*x*_Se-BSA is capable of up-regulating the hypoxia-inducible factor-1α (HIF-1α)/vascular endothelial growth factor (VEGF) pathway, facilitating wound recovery by stimulating mature collagen synthesis and supporting endothelial cell migration and proliferation [81]. Wang et al. [82] devised a dual-modal bactericidal strategy enhanced by acidic conditions, achieved through the integration of ROS generation and Zn^2+^ release into ZIF8/Au-GOx (ZAG) NPs. The hydroxyl radicals and Zn^2+^ work together to compromise bacterial membrane integrity and promote the leakage of intracellular contents, ultimately eliminating bacteria. More importantly, the production of gluconic acid in catalytic reactions enables local pH reduction to roughly 4.5 within the infected site, thereby enhancing both ROS generation and Zn^2+^ release efficiency, leading to superior bacterial eradication capability. You et al. [83] reported a micrometer-sized ferroferric oxide (Fe_3_O_4_)/MXene (FM) heterojunction. It is capable of releasing both ferric and ferrous ions, thereby inducing bacterial ferroptosis and effectively combating bacterial infection. Researches on the metal ion release-mediated synergistic bactericidal mode are listed in Table 5. Although the metal ion release-mediated bactericidal modality represents an effective strategy against bacterial resistance, challenges remain in precisely controlling ion release kinetics, minimizing potential cytotoxicity to host cells, and developing intelligent responsive systems for safer and more efficient applications.

**Table 5.** Metal ion-releasing-assisted nanozymes for antibacterial applications

Strategies	Nanozymes	Enzyme-like activities	Antibacterial mechanisms	Bactericidal performance	Ref.
Nanozyme + Metal ion release	CFO@MoS_2_	POD	Release of the Cu^2+^ + ·OH + SDT	Nearly 100% bactericidal rate against *S. aureus*	[170]
	OBG@CG	GOx	Release of the Cu^2+^ + ROS	Antibacterial rate (approximately 99%) against *S. aureus*	[171]
	Cu-MOF/GOx	GOx + POD	Release of the Cu^2+^ + GOx + ·OH	65% inhibition rates against *E. coli*, 80% against *S. aureus*	[172]
	FM heterojunction	CAT + SOD	Release of Fe^3+^ + ROS	For *E. coli*, ratio of dead cells 84.87 ± 3.15%	[83]
	MnS	OXD	Release of Mn_2_(S_3_)_3_ + ·O_2_^−^	Bactericidal efficiency approximately 80% against *S. aureus* and MRSA	[173]
	MoS_2_@CP	POD (K_m_TMB = 0.29 mM)	Release of the Cu^2+^ + ·OH + NIR	Excellent antibacterial against MRSA	[174]
	Ag/G-MoOx	OXD	Release of the Ag^+^ + ROS + NIR	Antibacterial efficacy up to 99.99% against MRSA	[175]
	CuTA NSs	SOD + CAT	Release of the Ag^+^ + ROS + bacterial-targeting aptamers	Bactericidal efficiencies are 98.51%, 97.17%, 93.13%, and 90.51% against *P. gingivalis*, *A. viscosus*, *S. aureus*, and *E. coli*, respectively	[176]
	ZIF8/Au-GOx	GOx + POD	Release of the Zn^2+^ + ·OH	MIC (4 μg/ml) for *S. aureus*	[82]
	Res@ZIF-67/Ce0.1Mn0.9-MMON	SOD + CAT + POD	Release of the Co^2+^ + PTT + ·OH	Bactericidal rates 97.4% of *E. coli* and 98.5% of *S. aureus*	[177]

### Immunity synergy-mediated bactericidal mode

Nanozymes enhance innate immunity by promoting phagocytosis and bacterial killing, and precisely target infection sites. Through temporal regulation, they exert potent bactericidal effects and promote antigen release in the early stage while clearing excess ROS and suppressing inflammation to facilitate tissue repair in the later stage. Meanwhile, nanozymes trigger an in situ vaccine effect, activating adaptive immunity and establishing long-term immune protection. The dual-edged sword effect of ROS and metal ions, together with the structure–activity relationships of nanozymes, determine immune enhancement or damage, providing a key strategy for synergistic anti-infection and tissue repair. Representative studies on nanozyme-mediated bactericidal effects assisted by immunity are summarized in Table 6.

**Table 6.** Immunity synergy-mediated nanozymes for antibacterial applications

Nanozyme	Enzyme-like activities	Antibacterial mechanisms	Immune target	Bactericidal performances	Ref.
ZnO-CuS	POD + GSH-Px + CAT	Positively charged anchoring + ROS-modulating + activate immunity	DC maturation	Strong destructive effect against MRSA biofilms	[178]
Ti-MnO_2_-CPA@Ce6	GOx + POD	ROS + SDT + activating adaptive immunity	Trained immunity	Bactericidal rate of *S. aureus* is 94%	[73]
SM@CuFeSe2	POD	The pathogen-treated membrane + NIR + ROS	Pathogen-treated membrane	Bactericidal rate of *S. aureus* is 100%	[86]
HBPL-MnO_2_	SOD + CAT	Inhibit the NF-κB, activate the Nrf2, and promote M2 macrophage polarization + NO	Macrophage polarization	Bactericidal rate is 94.1–99.5% of MRSA, *E. coli*, *P. aeruginosa*	[84]
FGO@MN	POD (K_m_H_2_O_2_ = 57.26 mM)	NIR + ferroptosis + reactivate neutrophil function	Reactivate neutrophil function	90% *S. aureus* biofilm elimination	[40]
MTF@MNs	POD	NIR + release Fe^2+^/Fe^3+^ + promote macrophage polarization toward pro-regenerative phenotypes	Macrophage polarization	(IC50) values are 54.54 μg for *S. aureus* and 48.73 μg for *E. coli*	[88]
ZIF-8@TA@MXene	POD + OXD	NIR + Zn^2+^ + ROS + immunomodulation	MAPK	Bactericidal rate of 99.9% against *E. coli*, *S. aureus*	[179]
CE	SOD + CAT (*K*_cat_ = 68.72μg/ml)	NIR + ferroptosis inhibition + induce macrophage M2 directional polarization + immune activating T cells number	macrophage polarization + immune activating	Bactericidal rate of 92.21% against *E. coli*, and 95.89% against *S. aureus*	[180]
PDA-PtCuTe	CAT	pH regulation + membrane damage + flagellar motility inhibition + scavenge ROS + macrophage polarization	Macrophage polarization	Survival rates of *E. coli* and *S. aureus* were (1.97 ± 0. 46)% and (2.83 ± 0.33)%	[181]
BIONPs	CAT	NIR + generate ^1^O_2_ + M1-macrophage phagocytosis	Macrophage polarization	High eradication efficiency in *E. coli* biofilm and *S. aureus* biofilm	[85]
HA@ MRuO_2_-Cip/GOx	GOx + POD (K_m_H_2_O_2_ = 0.13 mM)	ROS + trigger macrophage-related immunity	Macrophage polarization	Antibacterial efficiency of was 92.61%, 80.32%, and 70.2% for MRSA, *P. aeruginosa*, and *E. coli*, respectively	[90]
CPP NPs	SOD + CAT	aPDT + PS + scavenge extracellular ROS + macrophage polarization	Macrophage polarization	Strong biofilm penetration and excellent antibacterial efficiency against *P. gingivalis*	[87]

#### Immune potentiation

Nanozymes can reverse the immunosuppressed state of immune cells and activate the phagocytic and bactericidal functions of macrophages, neutrophils, and other innate immune cells to eliminate pathogens during antibacterial treatment. Biofilm-associated infections (BAIs) in wound tissues construct a dense biofilm structure and a high-ROS microenvironment, which severely suppress the immune response of resident immune cells, trigger immune cell dysfunction and adverse inflammatory reactions, and ultimately hinder wound healing. Therefore, single antibacterial strategies exhibit limited efficacy in eliminating refractory biofilm infections without synergistic immune repair. Nanozymes can remarkably reverse the immunosuppressive microenvironment of BAIs by disrupting biofilm architecture and catalytically scavenging excess ROS, thereby amplifying the innate immune response against biofilm infections. Studies have demonstrated that βNK@PtTe_2_ can dismantle compact biofilm structures via laser-triggered thermal driving force. After biofilm disruption and ROS elimination, NK@PtTe_2_ selectively promotes immune cell chemotaxis and activates conventional natural killer (cNK) cells to kill planktonic bacteria detached from disintegrated biofilms. In vivo*,* such immune microenvironment remodeling further facilitates the differentiation of cNK cells into long-lived tissue-resident NK (trNK) cells endowed with immune memory capability, effectively preventing secondary bacterial infection [84]. Moreover, iron ions are essential for bacterial metabolic homeostasis, which participate in the whole life processes of bacteria, including DNA synthesis, respiratory metabolism, virulence expression, and biofilm formation. Notably, bacterial biofilms can sequester iron ions from innate immune cells such as neutrophils, thereby restraining the host’s antibacterial immune response. Accordingly, nanozymes can enhance host immune defense and eliminate pathogens by regulating iron metabolism within the biofilm microenvironment (BME). The modulation of iron homeostasis not only disrupts dense biofilm structures and eradicates local infection but also rescues and reconstructs the impaired innate immune defense system. For instance, FGO@MN supplements iron ions in the BME, restoring the dysfunctional neutrophils. This strategy enhances the chemotaxis and phagocytic capacity of neutrophils and inhibits the excessive formation of neutrophil extracellular traps (NETs), further consolidating the elimination effect of biofilm infections [40]. Additionally, nanozymes are able to regulate macrophage polarization toward the pro-inflammatory M1 phenotype by catalytically generating ROS for bacterial eradication. As reported, bovine serum albumin (BSA) conjugated IrO_2_ nanoparticles (BIONPs) rebuild the host innate immune defense by facilitating M1 macrophage polarization, which efficiently phagocytose and eliminate residual bacteria, achieving prominent in vivo antibiofilm performance [85].

Bare nanozymes are readily cleared by blood circulation, exhibiting poor accumulation efficiency, off-target toxicity, and insufficient immune activation. After entering the bloodstream, unmodified nanozymes are recognized as exogenous invaders by the innate and adaptive immune systems and are subsequently cleared by the reticuloendothelial system, thereby shortening their in vivo working duration. Surface functionalization via biomimetic membrane integration of erythrocytes, platelets, and bacterial membranes into nanomaterials renders nanozymes resistant to immune clearance and capable of longer blood circulation. Dendritic cells (DCs) are critical antigen-presenting cells that play an indispensable role in innate and adaptive immune activation. Toll-like receptors (TLRs) abundantly expressed on DC membranes can specifically recognize pathogenic components, endowing DC-derived membranes with excellent immune evasion and targeted delivery capability. For example, bacteria-pretreated DC membranes were employed to encapsulate a (100) facet-oriented CuFeSe_2_ to construct the SM@CuFeSe_2_ nanosystem. Such a biomimetic design markedly enhances the specific binding affinity toward *S. aureus*, integrating immune evasion and bacterial targeting to achieve highly efficient bactericidal performance [86].

In summary, nanozymes improve innate immune clearance efficiency through 2 major immune-potentiating pathways. Nanozymes remodel the immunosuppressive BME and regulate ROS and metal ion metabolism, thereby restoring and activating the phagocytic and bactericidal functions of macrophages, neutrophils, and NK cells. Furthermore, biomimetic membrane modification enables inflammatory targeting and immune evasion, achieving precise immune activation at infectious sites. These 2 synergistic immune-enhancing mechanisms jointly accelerate pathogen elimination, providing an advanced immunotherapeutic strategy for refractory BAIs.

#### Immune tuning

ROS act as a double-edged sword in disease treatment. Bacterial elimination relies on robust ROS generation, whereas excessive ROS accumulation severely disturbs the inflammatory cascade and induces overactivation of endogenous immune cells, resulting in dysregulated macrophage polarization. Excessive pro-inflammatory macrophages and decreased reparative macrophage populations trigger excessive immune stimulation, which may further induce cytokine storms, inhibit fibroblast migration, delay angiogenesis, and aggravate soft and hard tissue damages. Therefore, the adverse effects of oxidative stress should be fully considered when utilizing nanozymes for antibacterial therapy. Exploring the temporal dynamic patterns of ROS generation, action, and elimination is critical for balancing bactericidal efficacy and inflammatory injury. It has been reported that CuTA-Por@ε-PL nanoparticles (CPP NPs) can efficiently eliminate bacterial biofilms through photodynamic-mediated ROS burst. Subsequently, the intrinsic SOD/CAT-like activities of CPP NPs convert redundant ROS into oxygen. Such a therapeutic pattern scavenges both inherent and residual ROS in periodontal tissues, alleviating intracellular oxidative stress in macrophages, regulating macrophage polarization, suppressing inflammatory responses, and facilitating tissue regeneration [87]. Similarly, the MTF@MNs microneedle system achieves staged antibacterial and immunomodulatory effects relying on ROS-related nanozymatic activities. At the initial stage of infected wound, the pathological microenvironment is characterized by low pH and excessive hydrogen peroxide. With the assistance of NIR irradiation and superior mechanical properties of microneedles, MTF@MNs effectively penetrate biofilm barriers and trigger iron-mediated Fenton reactions to generate cytotoxic hydroxyl radicals for high-efficiency antibacterial and antibiofilm performance. After the elimination of bacteria and biofilms, the wound microenvironment gradually returns to homeostasis, accompanied by neutralized pH and decreased local hydrogen peroxide concentration. At this stage, the inherent antioxidant capacities of MXene and tannic acid (TA) become dominant, removing residual ROS and suppressing inflammation to construct a regeneration-promoting immune microenvironment. This temporally responsive functional transformation enables MTF@MNs to achieve precise treatment by adaptively utilizing dynamic alterations of the pathological wound microenvironment [88].

In conclusion, nanozymes realize dynamic temporal conversion between ROS generation and elimination. In the early infectious stage, high levels of ROS ensure efficient bactericidal performance and immune recognition; in the later repair stage, inherent antioxidant capacities eliminate excess ROS, relieve excessive inflammation, and restore balanced macrophage to avoid chronic inflammation. Synergistically, these properties promote angiogenesis, collagen deposition, and re-epithelialization, achieving comprehensive immune modulation from antibacterial and anti-inflammation to tissue regeneration.

#### Adaptive immunity/immunoprotection

Chronic infectious diseases are generally characterized by immune cell exhaustion, elevated secretion of pro-inflammatory cytokines, and persistent bacterial colonization. Single regulatory strategies targeting innate immune cells (neutrophils, macrophages, etc.) exhibit limited efficacy in generating antibacterial adaptive immunity, failing to meet the long-term anti-infection requirements of chronic diseases such as diabetes mellitus (DM). In contrast, adaptive immune responses mediated by T and B lymphocytes can effectively eliminate residual bacteria in infected tissues and protect the host from recurrent pathogen invasion. It has been demonstrated that Ti-MnO_2_-CPA@Ce6 can induce explosive bacterial death and trigger the massive release of bacterial-associated antigens. The immunostimulatory Mn^2+^ further promotes the maturation of DCs, enhances antigen presentation efficiency, and activates T/B lymphocyte-mediated adaptive immunity to achieve long-term antibacterial capacity. In a rat implant-associated infection model, Ti-MnO_2_-CPA@Ce6 triggered systemic immune responses and induced durable T and B cell immune memory, thereby providing long-term defense against bacterial reinfection. In summary, nanozymes disrupt bacterial membranes and biofilm structures to induce bacterial lysis, which exposes abundant bacterial antigens to realize in situ vaccination. Moreover, metal ions and ROS-derived danger signals act as immune adjuvants to facilitate DC maturation and antigen presentation, activating T/B lymphocyte-dependent adaptive immunity and ultimately forming durable immune memory. This immunomodulatory strategy provides a sustainable immune protection approach for intractable chronic bacterial infections [73].

#### Regular pattern of immune synergy

ROS and metal ions exhibit typical dual-edged biological effects in infection immunomodulation, and the final immune outcomes are strictly governed by their dosage, spatiotemporal distribution, and action selectivity. Appropriate and spatiotemporally controllable ROS and metal ions achieve immune potentiation, facilitating pathogen clearance, immune cell activation, anti-inflammatory effects, and wound healing. In contrast, excessive, sustained, and nonselective release of ROS and metal ions induces immune disorders, triggers cytokine storms, and causes damage to normal soft and hard tissues. On this basis, nanozymes present definitive structure–activity relationships. The surface charge and modified ligands of nanozymes regulate the internalization efficiency and intracellular localization in immune cells. Relevant studies have reported that pattern recognition receptors (PRRs), particularly TLRs that identify pathogen-associated molecular patterns (PAMPs), are highly expressed on the membranes of bacteria-infected macrophages. Therefore, coating nanozymes with bacterial-infected macrophage membranes can effectively enhance bacterial capture capacity. Furthermore, catalytic selectivity and ROS types determine the immune regulatory tendency of nanozymes. OXD- and POD-like nanozymes trigger immune activation by inducing bacterial antigen release and promoting M1-type macrophage polarization, while CAT- and SOD-like nanozymes eliminate redundant extracellular ROS and facilitate M2 macrophage polarization to suppress excessive inflammation. Additionally, the degradable properties and ion release kinetics of nanozymes set the biological tolerance limit and build a stable immune safety range. In conclusion, the synergistic immune effects of nanozymes are comprehensively determined by physical structures, catalytic properties, and ion metabolism behaviors. Rational microstructure design and precise spatiotemporal regulation of ROS and metal ions enable multi-level synergistic immunomodulation, ranging from innate immune activation and inflammatory microenvironment remodeling to long-term adaptive immune protection. This work provides universal design principles for the exploitation and clinical translation of intelligent antibacterial nanomaterials.

### Cascade catalysis-mediated bactericidal mode

Nanozyme cascade reactions refer to orderly coupled catalytic reaction sequences centered on nanozymes, where the product of the preceding reaction serves as the substrate for the next step. This biomimetic design boosts catalytic activity, realizes multi-function integration, and achieves spatiotemporal regulation, showing great potential in biomedicine, environmental treatment, and biosensing fields.

Localized hyperglycemia in diabetic wound infections contributes to the characteristic pathological manifestations of diabetic ulcers, including chronic secondary infections, compromised angiogenesis, oxidative stress, and diabetic peripheral neuropathy. POD-like nanozymes represent the most widely used antibacterial agents, which convert H_2_O_2_ into ·OH to combat bacterial infections. In light of this catalytic principle, the generation of adequate ·OH generally depends on external H_2_O_2_ combined with acidic conditions (typically pH 3.0 to 6.0) for most POD-like nanozymes. In fact, although inflammatory immune cells at infected sites secrete abundant H_2_O_2_, bacteria and their biofilms themselves produce negligible amounts of H_2_O_2_. Diabetic wound microenvironments only maintain mild acidity overall, which fails to support continuous high-yield ·OH production and often leads to incomplete bacterial elimination. Furthermore, untargeted nanozymes with constitutive catalytic activity tend to damage normal cells via off-target ROS generation and deliver unsatisfactory antibacterial outcomes. Moreover, the dosage of exogenous H_2_O_2_ is challenging to regulate precisely and is susceptible to decomposition, potentially leading to damage to healthy tissues and disruption of the wound microenvironment [89]. Therefore, GOx, which binds glucose and catalyzes its oxidation into gluconic acid and hydrogen peroxide, represents a promising candidate to remodel the hyperglycemic microenvironment of diabetic wounds. Beyond generating catalytic substrates (H_2_O_2_) and lowering local pH via gluconic acid production, GOx continuously consumes excess glucose within lesion sites to mitigate hyperglycemia, thereby relieving the inhibitory effect of high glucose on angiogenesis and accelerating tissue repair. Nevertheless, excessive GOx dosage may overdeplete physiological glucose around normal tissues, disrupt the metabolic homeostasis of healthy cells, and trigger off-target cytotoxicity, which restricts in vivo application at high loading concentrations [90]. GOx catalyzes the transformation of glucose into H_2_O_2_, which then acts as the substrate to produce hydroxyl radicals. Moreover, the gluconic acid generated can reduce the local pH, which in turn enhances hydroxyl radical production, given that POD-like nanozymes generally display greater catalytic activity in acidic environments. Research has demonstrated that glucose-responsive photothermal nanozymes (GOx/MPDA/Fe@CD) can penetrate bacteria, where they catalyze glucose to generate H_2_O_2_ and create an acidic microenvironment. This promotes Fenton reaction-mediated POD-like catalytic activity, leading to the production of toxic ·OH. This cascade amplifies Fenton-mediated POD-like activity to yield cytotoxic ·OH, and such ROS-dependent CDT therapy achieves thorough bacterial clearance [73,89].

GSH is widely found in Gram-negative bacteria and Gram-positive bacterial species. The microenvironment surrounding bacterial infections generally displays abnormally high levels of GSH. Under oxidative stress, GSH is oxidized to GSSG to consume excess ROS [91]. GSH functions as a key endogenous antioxidant across biological environments and efficiently scavenges intracellular ROS. As a promising alternative to conventional antibiotics, the utilization of Fenton-type reactions to decompose H_2_O_2_ and produce ·OH has attracted considerable interest in recent years, owing to its distinctive oxidative antibacterial activity and minimal risk of resistance. However, redox homeostasis—an intrinsic cellular defense system— greatly restricts CDT-mediated antibacterial therapy by preserving the equilibrium between oxidants and antioxidants. At infection sites, the overexpression of GSH, a primary cellular antioxidant, can effectively lower ROS abundance and protect against free radical-induced oxidative harm, leading to obvious resistance against a wide range of ROS-based therapies. Increasing evidence suggests that modulating H_2_O_2_ levels and depleting GSH are effective strategies for enhancing ROS-induced oxidative damage in bacterial cells. Therefore, nanozymes can exert bactericidal effects by depleting GSH. Studies have shown that MOF-modulated Fe_3_O_4_ composite Au NPs can continuously convert oxygen into highly toxic ·OH through GSH-depleting cascade redox reactions, enabling CDT-mediated bacterial eradication and treatment of bacterial-infected wounds [50]. Other cascade catalysis-mediated bactericidal modes for nanozymes are listed in Table 7.

**Table 7.** Cascade catalysis-mediated nanozymes for antibacterial applications

Nanozymes	Enzyme-like activities	Antibacterial mechanisms	Bactericidal performances	Ref.
Cu/Bro/Gox	GOx + POD (K_m_H_2_O_2_ = 1.95 mM)	·OH + Cu^2+^	Bacterial survival rates were 0.82% for *S. aureus* and 2.57% for *E. coli*	[182]
GFeF	GOx + POD	·OH	Efficient bactericidal activity against *E. coli* and *S. aureus*	[183]
FCSGP	GOx + POD	·OH + CDT + PTT	89.79% biofilm inhibition against *S. aureus* and *C. albicans*	[74]
QC-OD@AF/S	GOx + POD	·OH + quaternary ammonium groups	Excellent antibacterial activity against *S. aureus* and *E. coli*	[184]
Fe2C/GOx@MNs	GOx + POD	·OH	Eliminated 93.21% ± 0.37% MRSA biofilm	[185]
GOX/MPDA/Fe@CDs	GOx + POD	·OH + PTT + CDT	Survival rate of MRSA was close to ∼0%	[89]
Cu@ZIF/GOx	GOx + POD	·OH	Inactivate 6.33 log of *S. aureus*, and 6.44 log of *E. coli*	[186]
GOx-OsNCs	GOx (91.2 U mg^−1^) + POD + CAT	·OH	Excellent antibacterial activity against *E. coli* and *S. aureus*	[187]
L-Arg/GOx@TA-Fe(III)	GOx + POD (K_m_H_2_O_2_ = 3.02 mM)	ROS + NO	The survival rate of *E. coli* and MRSA was nearly decreased to 20%	[188]
MnFe_2_O_4_@MIL/Au&GOx	GOx + POD (K_m_H_2_O_2_ = 35.67 mM)	ROS + GSH consumption	Bacterial survival rates of *E. coli* and *S. aureus* were 0%	[91]
PDA/Fe_3_O_4_	POD (K_m_H_2_O_2_ = 0.597 mM)	ROS + GSH consumption + PTT	The antibacterial efficiencies reached 97.4% for *S. aureus* and 90.1% for *E. coli*	[50]
BioSAzyme	HRP + GSHOx + GPx	Localize the biofilm glycocalyx + ·OH + GSH consumption	Bacterial survival rate in the *E. coli* biofilm is 0.36%	[189]

### Microenvironment regulation-mediated synergistic bactericidal mode

At present, novel treatment modalities such as PTT, PDT, CDT, and gas therapy are being applied to treat bacterial wounds, with satisfactory results achieved. However, such single-modal therapies often show low bioavailability in the absence of nanocarriers, whereas optimized nanodelivery platforms greatly boost their local accumulation at infection sites. Stimuli-responsive nanozymes have been engineered for the treatment of drug-resistant bacteria-infected wounds as shown in Table 8. In response to external or internal stimuli, these materials can achieve on-demand antibacterial effects by releasing bactericidal agents or altering their own physicochemical properties, thereby demonstrating considerable future application potential. For instance, Yang et al. [92] reported a hydrogel wound dressing strategy featuring bacteria-responsive self-activating antibacterial properties and multiple nanozyme activities, which effectively remodeled the regenerative microenvironment and accelerated infected wound healing. These hydrogels respond to multiple factors within the bacterial metabolic microenvironment, enabling on-demand antibacterial action and biofilm elimination through the conversion of bacterial metabolites, along with CDT enhanced by nanozyme activity in combination with self-driven nitric oxide (NO) release. This composite hydrogel displayed “self-diagnostic” capabilities for detecting and responding to variations in the wound microenvironment. Wu et al. [93] reported a Cu/Mn-co-doped ZnO tandem nanozyme (ZnO-CM) featuring pH-responsive multienzyme-mimicking activities. Under acidic conditions, its POD-like activity facilitates the conversion of H_2_O_2_ into highly toxic ·OH, thereby demonstrating potent antibacterial efficacy. In a neutral pH setting, its SOD- and CAT-like functions effectively remove excessive ROS, elevate antioxidant capability, and mitigate oxidative stress, thus relieving colitis caused by Salmonella infection. Jiang et al. reported a Ca^2+^-crosslinked sodium alginate (SA) and chitosan (CS) hydrogel containing Mo,Fe/Cu,I-Ag loaded with GOx (Mo,Fe/Cu,I-Ag@GOx nanozyme gel), which exhibits multi-enzyme-like activities. The nanozyme gels displayed intrinsic GOx, POD-like, OXD-like, CAT-like, and SOD-like activities, enabling a pH-switchable glucose-initiated cascade reaction for diabetic wound healing. In the first step of the cascade, driven by GOx, the nanozyme gel facilitates the transformation of glucose and O_2_ into gluconic acid and H_2_O_2_, leading to ·OH production that kills pathogenic bacteria. In the second cascade reaction, as the wound microenvironment shifts to an alkalescent pH, the nanozyme gel mimics SOD to catalyze the conversion of ·O_2_^−^ into O_2_ and H_2_O_2_. Subsequently, its CAT-like activity decomposes both endogenous and exogenous H_2_O_2_ into O_2_, thereby reducing oxidative stress and alleviating hypoxia [81]. Although this strategy demonstrates broad application prospects, its implementation still faces a series of critical challenges. For example, stimulus signals such as decreased pH and local H_2_O_2_ in infected microenvironments are weak and unstable. Wound pH varies greatly: Mild infections drop pH from 7.4 to ~6.5, severe suppurative wounds reach pH 5.0 to 6.0, and chronic wounds stay neutral or weakly alkaline. Such unstable stimuli reduce material responsiveness and weaken bactericidal reliability. Table 8 summarizes pH-responsive nanozymes for antibacterial applications.

**Table 8.** Microenvironment-regulated nanozymes for antibacterial applications

Strategies	Nanozymes	Enzyme-like activities	Antibacterial mechanisms	Bactericidal performances	Ref.
pH-responsive	MSCO	SOD + CAT	ROS + NO + Cu^2+^ release	Antibacterial efficiencies against MRSA is 99.2%	[92]
	FPB-Co-Ch NPs	SOD + CAT + POD + OXD	Electrostatic adsorption + cascade reactions	Highly efficient bactericidal activity against *H. pylori*	[121]
	Mo,Fe/Cu,I-Ag@GOx	GOx + POD (K_m_TMB = 0.53 mM) + OXD + CAT + SOD	Cascade reactions	Effectively inhibited the formation of *S. aureus* and *P. aeruginosa* biofilms	[81]
	ZnO-Cu/Mn	POD + SOD + CAT	Cascade reactions	Bactericidal efficiency of 98% against *Salmonella*	[93]
	CSG-Mx	POD (K_m_H_2_O_2_ = 0.22 mM) + OXD	ROS	Antibacterial efficiencies are 95.25% for *S. aureus* and 100% for *E. coli*	[190]
	CeO_2_	GOx + CAT	Release of the Cu^2+^ + ROS elimination	Antibacterial efficacy was over 99% inhibition rates against *S. aureus* and *P. aeruginosa*	[191]

Infection microenvironments should be regarded as dynamic and heterogeneous catalytic constraints rather than static triggers. First, pH varies across tissue sites, infection stages, and biofilm depths. Although acidic microenvironments can activate many POD-like nanozymes, chronic wounds, mature biofilms, and certain mucosal infections may exhibit neutral or weakly alkaline conditions, causing acid-dependent ROS-generating systems to lose efficiency. Second, hydrogen peroxide is often present at low and spatially heterogeneous levels, which limits sustained POD-like and Fenton-type catalysis. Third, oxygen availability is restricted in dense biofilms and poorly vascularized tissues, thereby reducing OXD-like catalysis and other oxygen-dependent reactions. Fourth, bacteria and host cells compete for oxygen and nutrients, and bacteria may adapt metabolically by entering dormant states, slowing growth, reinforcing biofilm matrices, or activating oxidative stress defense systems. These adaptations can reduce nanozyme susceptibility and weaken rapid catalytic killing.

Host immune cell interactions further reshape infection microenvironments. Neutrophils and macrophages can locally alter ROS levels, cytokine profiles, protease activity, metal ion availability, and pH, which may either enhance nanozyme-mediated pathogen clearance or aggravate inflammatory tissue injury. In addition, bacterial EPS, GSH, serum proteins, and host antioxidants can consume reactive species, passivate active sites, or modify nanozyme–bacteria interactions. Therefore, microenvironment-responsive nanozymes should not be designed to respond to a single trigger only. Instead, future systems should broaden the pH-response window, self-supply H_2_O_2_ or O_2_ when necessary, deplete excessive GSH, penetrate or degrade EPS barriers, and coordinate antibacterial activity with immune and tissue-repair processes.

Accordingly, future work should improve stimulus sensitivity, expand responsive windows, and construct smart antibacterial platforms adaptable to complicated pathological microenvironments.

### Other bactericidal modes

Compared with other antimicrobial strategies, ultrasound has garnered considerable interest owing to its excellent tissue penetration and biocompatibility. Emerging ultrasound-responsive nanotherapies have arisen as a transformative strategy for tackling multidrug-resistant infections and facilitating tissue repair. Among these strategies, SDT has become a prominent noninvasive approach that employs ultrasound-activated sensitizers to produce cytotoxic ROS via precisely controlled cavitation, mechanical forces, and thermal effects. By disrupting bacterial metabolism and inducing DNA damage, this process ultimately induce bacterial death. However, systemic safety concerns—particularly regarding long-term in vivo retention—remain unresolved.

In addition to the bactericidal strategies discussed above, target-modified nanozymes have been specifically designed for more precise and safer bacterial elimination. In recent studies, we have categorized 3 types of targeting strategies: Nanozymes themselves can possess bacterial-targeting capacity through enzymatic activity or electrostatic attraction. Small-molecule modified nanozymes can specifically recognize bacterial biomarkers. Typical ligands include CPB, mannose, dextran, chitosan, antimicrobial peptides, and aptamers, which bind bacteria via diverse patterns: Phenylboronic acid forms covalent bonds with bacterial polysaccharides, mannose undergoes receptor–ligand binding, and chitosan adheres to bacteria through electrostatic and hydrogen-bond interactions. Carrier-coated nanozymes can also achieve targeting. Studies have shown that PAA-Cnp phospholipid nanozymes inherently possess targeting capabilities, enabling them to bind to phospholipid components on bacterial cell membranes and target bacteria within biofilms [34,94,95]. PtCo@G@CPB GO nanozymes, modified with CPB molecules on their surface through hydrophobic interactions, can reversibly bind to peptidoglycan on bacterial cell walls and thereby achieve specific capture of *Helicobacter pylori* [96,97]. The biomimetic POD-like nanozyme (Co@SAHSs@IL-4@RCM) is fabricated by coating Co@SAHS with RAW264.7 macrophage cell membranes (RCMs). RCM firstly binds inflamed vascular endothelial cells to accumulate nanozymes at infection sites, and further directly recognizes bacteria via inherent membrane adhesion proteins, realizing dual-targeted bacterial elimination [98,99]. Related research studies on targeted modification-mediated bactericidal mode for nanozymes are listed in Table 9.

**Table 9.** Targeted modification-mediated nanozymes for antibacterial applications

Strategies	Nanozymes	Enzyme-like activities	Antibacterial mechanisms	Bactericidal performances	Ref.
Toll-like receptor	S-MM@CeO_2_-TCPP	POD (K_m_H_2_O_2_ = 3.86 mM)	Toll-like receptor anchoring + ROS + PDT	Moderate bactericidal activity against *E. coli*, *S. aureus*	[162]
HMVs	BiPt@HMVs	POD + OXD	HMVs + ROS + US	Highly effective against *E. coli* EC1322	[192]
RCM	Co@SAHSs@IL-4	POD (K_m_H_2_O_2_ = 0.81 mM) + GSH-PX (K_m_GSH = 0.19 mM)	RCM + ROS + PTT	Surviving colonies reduced by 99.8% and 98.0% against *E. coli* and *S. aureus*, respectively	[98]
ConA	BioSAzyme	HRP + GSHOx + GPx	Localize the biofilm glycocalyx + ROS + GSH consumption	Survival rates in the biofilms are 0.36% against *E. coli* and 1.47% against MRSA	[189]
PLC	PAA-Cnp	PLC (*K*_m_ = 171.8 mM)	Target bacterial cells inside the biofilms + phospholipase activity-based cell membrane	80% biocidal activity against *Salmonella*, thickness of the biofilm was drastically reduced	[34]
Phenolic hydroxyl in TA	TA-Fe/Cu NPs	POD	Hydrogen-bonding interactions with peptidoglycan + ROS	Bacterial eradication rate of at least 99% against *E. coli* and MRSA; PTT + nanozyme can effectively eliminate biofilm	[97]
Boronic acid	PtCo@G@CPB	OXD	Bind peptidoglycan	Notable bactericidal activity against *H. pylori* under gastric acid conditions	[96]
Cu^2+^- peptide bond	CuPB	OXD + POD + GPx	Inhibits ATP-binding cassette (ABC) transporters + ROS	*E. coli* viability was almost entirely suppressed at 200 μg ml^−1^	[193]
Dextran	Dex-IONP-GOx	GOx + POD	Target glucan-binding proteins + ROS	Antibiofilm activity results from the oxidative damage caused by ROS generation	[194]
Antibacterial peptide	Ag@Pt-Au-LYZ/HA-LL-37 NPs	GOx + POD + OXD	Target the bacterial cell membrane + ROS	MIC (*S*. *aureus*) = 8 μg/ml, MIC (*E*. *coli*) = 32 μg/ml	[195]
Phage	Phage@Pd	POD	Adhere to or wrap around bacteria + ROS	99.4% bactericidal efficiency against *E*. *coli* biofilm	[99]

Besides, enhancing bacterial adhesion capacity is also crucial for achieving highly efficient antibacterial activity in nanozyme-based antimicrobial agents [100]. Natural biological structures and functions originate from long-term evolutionary optimization, making them difficult to replicate or mimic with artificial materials. For example, the ability of viruses to infect host cells is mediated by spike proteins located on their envelopes. Inspired by nature, the assembly of biomimetic antibacterial nanozymes follows the principle that the morphology of nanozymes determines their surface structure and intermolecular interactions, thereby influencing catalytic performance. Nanozymes with intrinsic spiky structures can interact more directly with bacteria, which is highly beneficial for enhancing bactericidal performance. The impact of spikes on nanozymes is mainly reflected in their structural design, as this configuration helps improve the adhesion of nanozymes, thereby enhancing their bactericidal efficacy. Studies have shown that ICG@hMnOx nanozymes utilize a virus-spike surface for bacterial adhesion as a membrane-anchored ROS generator, thereby achieving bacterial capture [75]. NiCo_2_O_4_ nanozymes are capable of capturing bacteria with diverse morphologies through physical and mechanical interactions occurring between their nanostructures and the bacterial cells [101]. However, both rough-surfaced and spiky noble metal nanostructures—such as gold nanoflowers and nanostars—typically possess relatively large dimensions (>100 nm), resulting in greatly lower atomic utilization efficiency compared to ultrasmall gold NPs (~2 nm), which inevitably compromises their catalytic activity. Consequently, developing effective strategies to simultaneously enhance bacterial capture while retaining high intrinsic catalytic activity of ultrasmall noble metal NPs holds great promise for future nanozyme design. To address this, Feng et al. [94] developed a hybrid nanozyme construction approach that involves confining ultrasmall noble metal NPs within a lysozyme amyloid nanofibril network. Drawing inspiration from the bacterial entrapment strategy of human defensin 6 (HD6), which prevents microbial pathogen invasion of host cells through a tangled nanofiber network, the hierarchical amyloid-like supramolecular nanofiber web (HSNW) directs the formation of small metal crystals and retains catalytic activity on par with isolated metal NPs. Leveraging this intrinsic supramolecular network, the LNF/Aum Cun HSNW system achieves efficient bacterial capture and promotes localized catalytic attack around the surfaces of bacterial cells.

## Antimicrobial Applications of Nanozymes

Infectious diseases caused by bacteria, fungi, and viruses pose persistent threats to public health. As enzyme-mimicking catalytic materials, nanozymes have been explored across diverse antimicrobial interfaces, including abiotic surfaces, superficial tissues, deep-seated infections, fungal infections, and viral inactivation. Although these applications share certain catalytic principles, such as oxidant generation, hydrolytic disruption, metabolic interference, and immune modulation, their design requirements differ substantially according to pathogen type, infection depth, tissue interface, delivery route, and local microenvironment.

From an application-driven perspective, antibacterial nanozyme design should be considered across 3 representative levels. Abiotic interfaces, including marine equipment, coatings, and medical devices, require long-term catalytic durability, antifouling capability, and low environmental toxicity. Superficial and locally accessible infections, such as wounds, dental biofilms, bacterial vaginosis (BV), and keratitis, emphasize local retention, biofilm penetration, controllable activation, and tissue compatibility. Deep-seated infections, including pneumonia, gastritis, enteritis, sepsis, abscesses, and implant-associated infections, impose stricter requirements for biodistribution, delivery route, microenvironment-responsive activity, biodegradation, and systemic safety. This application-driven logic provides the basis for organizing the representative examples summarized below.

### Antibacterial applications

#### Marine equipment biofouling control

**Fig. 6.**
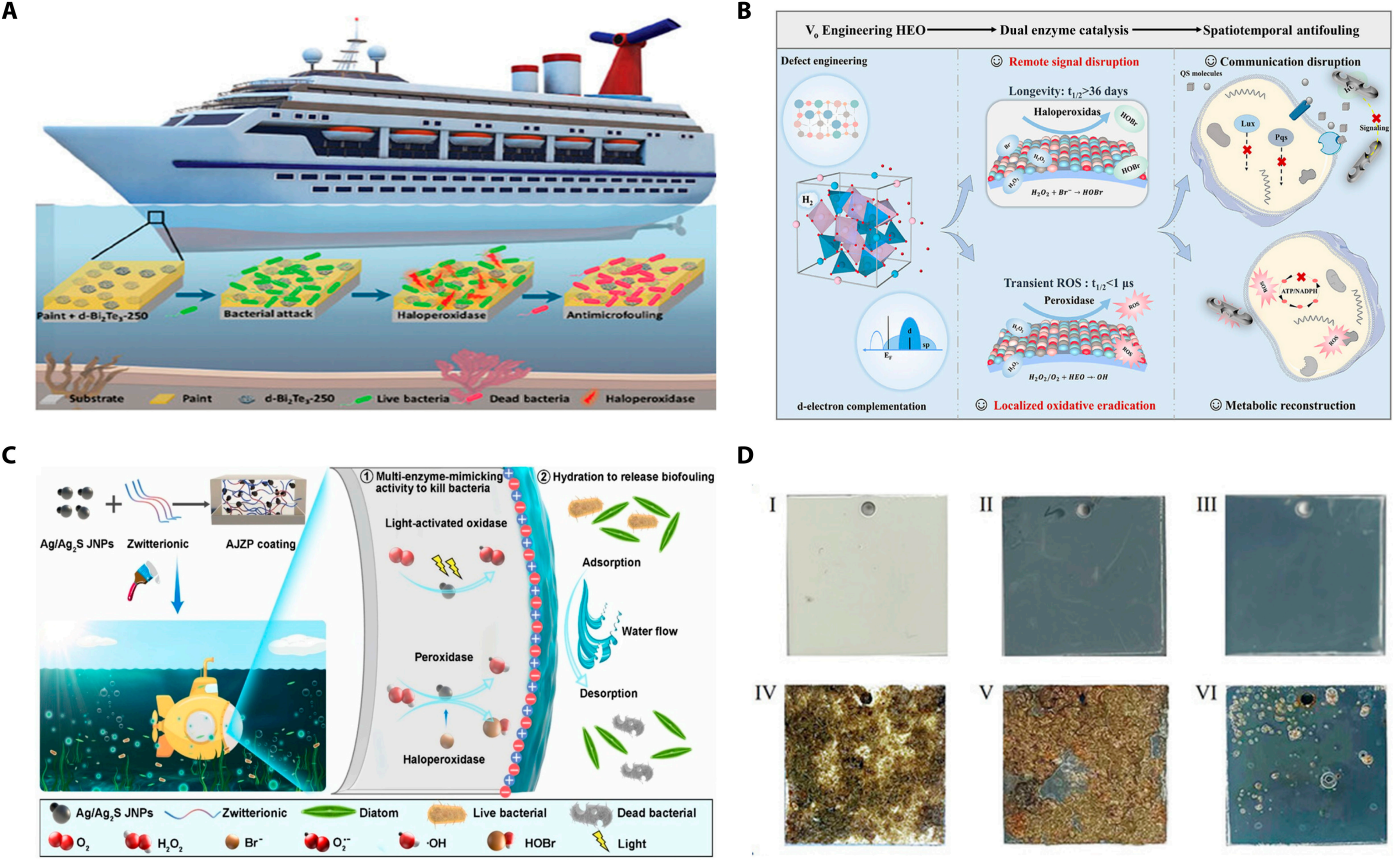
Application of nanozymes in instrument antibacterial. (A) Representative schematic of a ship’s hull coated with conventional paint mixed with the d-Bi_2_Te_3_-X nanosheets for enhanced haloperoxidase, antibacterial, and antimicrofouling activity [104]. Copyright 2024, Wiley-VCH Verlag. (B) Schematic diagram of the antifouling mechanism of V-HEO nanozymes [105]. Copyright 2026, Wiley-VCH Verlag. (C) Schematic of the synergistic antifouling mechanism and marine evaluation of Ag/Ag_2_S Janus nanoparticles (AJZP) coating [106]. Copyright 2024, Elsevier. (D) Digital images of stainless-steel coating panels with or without additives after 62 d of the static field immersion in open ocean, fresh panels: (I) blank, (II) N/C, and (III) Mo SA-N/C; fouling panels: (IV) blank, (V) N/C, and (VI) Mo SA-N/C [107]. Copyright 2022, Wiley-VCH Verlag GmbH.

Marine biofouling is defined as the colonization and proliferation of marine organisms—including bacteria, algae, and barnacles—on the surfaces of marine artificial structures. This phenomenon increases hull drag, accelerates surface corrosion and propeller wear, and further raises fuel consumption and routine maintenance costs. The initiation of biofouling involves the adhesion of prokaryotic microorganisms, such as bacteria, to preexisting organic films. These microorganisms subsequently secrete EPS, forming a microbial matrix that promotes the settlement of additional fouling organisms, ultimately compromising the integrity of marine infrastructure [102]. In addition to traditional physical, chemical, and biological methods, research has found that natural enzymes can inhibit bacterial adhesion through the hydrolysis of proteins and carbohydrates, achieving anti-fouling performance comparable to commercial coatings [103]. Certain marine algae, such as *Corallina officinalis*, inhibit biofilm formation through the secretion of HPO. The 2-electron oxidation of halides to microbicidal hypohalous acids (HOX, X: Cl^−^, Br^−^, I^−^) or analogous oxidized halide species is catalyzed by these natural enzymes in conjunction with H_2_O_2_. Leveraging naturally occurring reagents such as halides and H_2_O_2_, this mechanism constitutes an effective and environmentally benign antibiofilm strategy. Consequently, coating marine equipment with CeO_2_@ZrO_2_ can inhibit biofilm formation due to its HPO-mimicking catalytic activity [103]. In another study, manganese selenide nanoflowers (MnSe NFs) were introduced as broad-spectrum antibacterial nanozymes, which inhibit over 99.999% of both Gram-positive and Gram-negative bacteria through their phosphoesterase-, OXD-, and POD-like activities. Furthermore, coatings based on MnSe NFs were shown to markedly suppress biofilm formation on ship hulls for more than 90 d under marine antifouling conditions [103]. Recent studies have demonstrated the potential of defect-engineered nanomaterials as artificial nanozymes mimicking HPO activity, tackling long-standing marine biofouling issues. Firstly, defect-engineered bismuth telluride (Bi_2_Te_3_) nanosheets, acting as artificial nanozymes, drastically enhance hypohalous acid production, exhibiting up to an 8-fold improvement in antibacterial and antimicrofouling performance (Fig. 6A) [104]. Another study also introduced multifunctional nanozymes, oxygen vacancy-enriched high-entropy oxides (Vo-HEO), inspired by HPO, which efficiently generate hypobromous acid (HOBr) mimicking HPO activity. These Vo-HEOs overcome key challenges in marine antifouling by disrupting bacterial quorum sensing and reducing bacterial adhesion by 90%, offering a novel approach for adaptive antifouling design and intelligent regulation of marine microecology (Fig. 6B) [105]. Additionally, a transparent zwitterionic coating based on Ag/Ag_2_S Janus NPs (Ag/Ag_2_S JNPs), which combines POD, light-activated OXD, and HPO-mimicking activities, and generates reactive species such as hydroxyl radicals, HOBr, and superoxide radicals, can effectively combat biofouling. The zwitterionic coating further enhances antifouling capacity by forming hydration layers, demonstrating a 74.91% reduction in fouling after 90 d of marine immersion (Fig. 6C) [106]. By photothermal strategy, Mo SA-N/C nanozyme can mimic HPO activity and accelerate the formation of hypobromous acid under light. This nanozyme exhibits strong antibacterial activity and effectively inhibits biofouling in marine environments (Fig. 6D) [107]. Collectively, such HPO-mimicking nanozymes possess great application potential for eco-friendly and cost-effective marine antifouling therapy.

#### Superficial infection

Skin breakage and wound formation render the wound microenvironment vulnerable to bacterial infection, among which chronic wounds face particularly high infection risks, represented by diabetic skin ulcers, severe burns, and scalds. Once contaminated by bacteria, these wounds show impaired healing capacity and need prolonged clinical intervention [108]. If treatment fails to control infection, severe clinical complications may occur.

Infected wounds are difficult to heal mainly due to bacterial biofilm formation [94,109]. Biofilms utilize EPS produced by bacteria as physical and chemical barriers, obstructing antibiotic penetration and compromising the host immune response [110]. EPS mainly consists of proteins, eDNA, and polysaccharides, forming a unique microenvironment inside biofilms featured with hypoxia, localized weak acidification (pH ranging from 5.0 to 6.2), and abundant reduced GSH. GO-based NTA-Ce(IV) composite materials (GO-NTA-Ce) can effectively inhibit biofilm formation and disperse established biofilms by degrading eDNA, thereby eradicating antibiotic-resistant bacterial biofilm infections. Targeted disruption of key EPS components further prevents biofilm formation [33]. Ferumoxytol, which exhibits high POD-like activity at acidic pH, kills bacteria in situ by disrupting the bacterial membranes and degrades the EPS matrix through the cleavage of glucan structures. This process prevents the accumulation of cariogenic biofilms and protects the enamel surface from damage, thereby inhibiting the development of severe dental caries. Furthermore, its activity diminishes at near-neutral (physiological) pH, and it does not produce harmful side effects in vivo [111]. Additionally, the abundant GSH within biofilms can be exploited. For example, the biomimetic single-atom nanozyme Co@SAHSs@IL-4@RCM with dual GPx- and POD-like activity is coated with RAW264.7 macrophage membranes. It can selectively accumulate at biofilm-infected wound sites, deplete intracellular GSH to disrupt BME, eliminate pathogenic bacteria, and ultimately accelerate the repair of infected wounds [98].

**Fig. 7.**
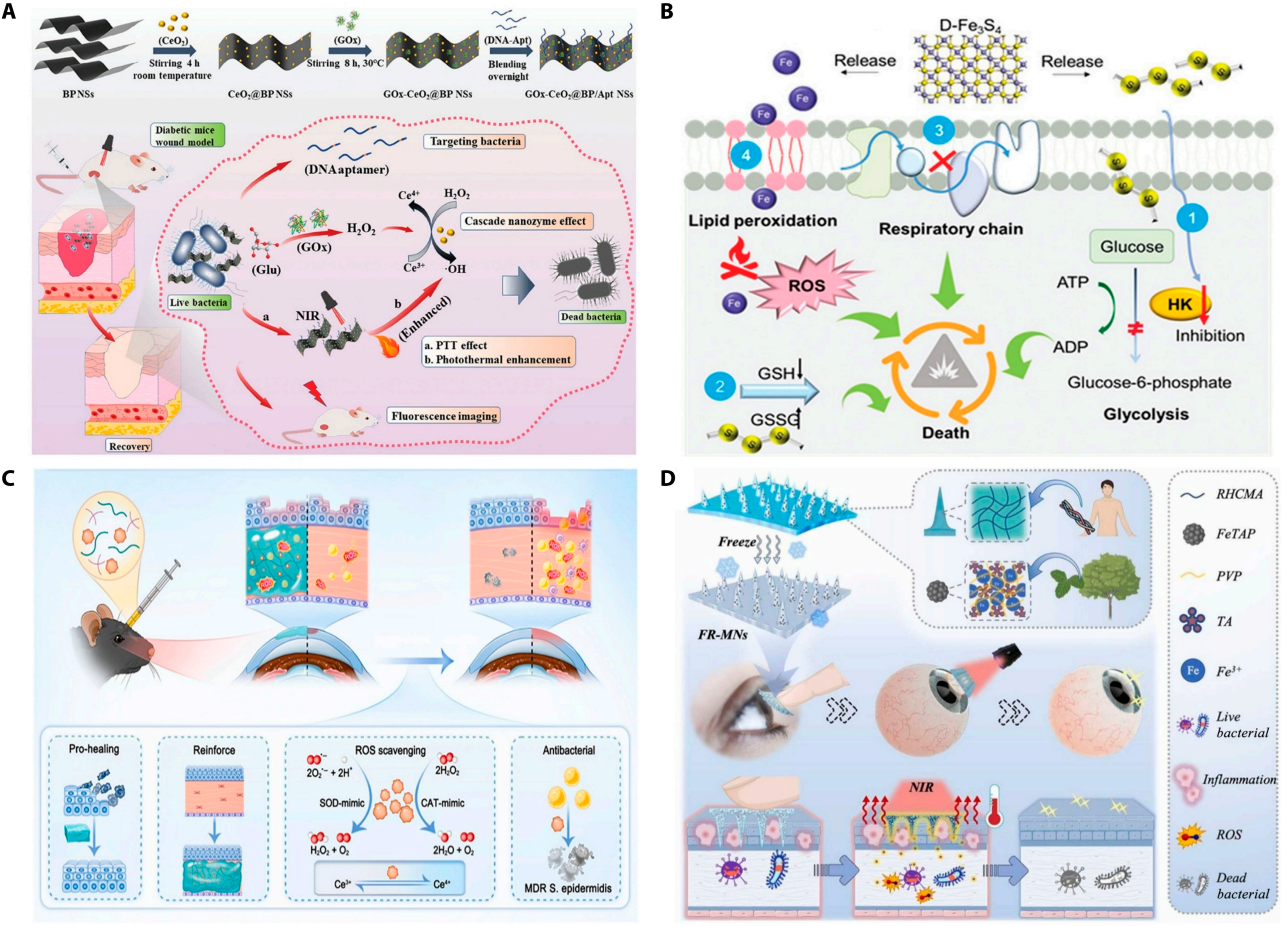
Application of nanozymes in superficial infections. (A) Schematic depiction of the preparation and application of GOx-CeO_2_@BP/Apt in diabetic infected wound. BP NSs, black phosphorus nanosheets [113]. Copyright 2025, Wiley-VCH. (B) Schematic overview of *G. vaginalis* death mechanisms via iron polysulfide-triggered ferroptosis [115]. Copyright 2022, Wiley-VCH Verlag. (C) Schematic illustration of the synthesis of ANHD and its therapeutic use for BK [116]. Copyright 2026, Elsevier. (D) Schematic diagram of the FR-MNs and their application for treating keratitis [117]. Copyright 2023, Elsevier.

Diabetic ulcers, unlike conventional wounds, are characterized by high concentrations of glucose. Studies have shown that high glucose concentrations can promote the self-sufficiency of H_2_O_2_, thereby overcoming the threshold of H_2_O_2_ concentration in the physiological state [112]. This mechanism furnishes a novel approach for the utilization of H_2_O_2_ during diabetic ulcer repair and may represent an effective strategy for enhancing diabetic wound healing. DNA aptamer-targeted modification of GOx and cerium oxide nanosheets (GOx-CeO_2_@BP/Apt) simultaneously exhibits both POD-like activity and GOx-like activity (Fig. 7A) [113]. By leveraging the high glucose concentration in diabetic mice, this system enables self-supply of H_2_O_2_ and continuous production of ·OH. Moreover, by integrating NIR light responsiveness and DNA-targeted properties, this system can effectively destroy the cell membranes of *S. aureus* and *E. coli*, with corresponding MICs of 2 and 16 μg/ml (BP content included), respectively. Owing to its cascade catalytic behavior, the system surmounts the constraints of H_2_O_2_ under physiological conditions, thus effectively facilitating the healing of infected diabetic wounds.

In addition to conventional superficial infections (Table 10), BV is also among the types of superficial infections urgently requiring new alternative antimicrobial agents. The pathogenesis involves dysbiosis, where the reduction of lactobacilli leads to the overgrowth of other pathogens such as *Gardnerella vaginalis*. Common clinical treatments include oral or topical antibiotics such as metronidazole. However, Gardnerella is highly prone to developing resistance to metronidazole, resulting in over half of patients experiencing recurrence within a year [114]. Gao’s team [115] discovered that metastable iron sulfide (mFeS) possesses HPO-like activity and kills bacteria in a Gram-dependent manner. The antibacterial activity is most effective against Gram-variable Gardnerella species > Gram-negative bacteria >> Gram-positive bacteria. Among them, Fe_3_S_4_ demonstrated the strongest antibacterial capability, effectively inhibiting the growth of *G. vaginalis* and partially inhibiting *E. coli* while having no inhibitory effect on Gram-positive bacteria (such as *S. aureus* and *Lactobacillus*). The MIC against *G. vaginalis* is 7.8 μg/ml, with an antibacterial effect superior to that of metronidazole. The preventive and therapeutic effects of Fe_3_S_4_ suspension and its suppository on BV in mice was further evaluated using a mouse vaginitis model, and the results showed that both dosage forms could markedly reduce the number of *G. vaginalis* in the vagina of mice, indicating that metastable iron sulfide could be promising non-antibiotic drugs for the prevention and treatment of BV (Fig. 7B). For the treatment of bacterial keratitis (BK), numerous studies have developed various nanozyme-based delivery systems to boost therapeutic efficacy. One representative candidate is the injectable antibiotic-nanozyme hydrogel depot (ANHD), fabricated by integrating Ce-gatifloxacin nanozymes (CGNs) into xanthan–polyethylene glycol (PEG) hydrogel. This composite exerts triple functions: broad-spectrum antibacterial activity, ROS scavenging, and wound healing promotion. It strengthens corneal tissue without penetrating the corneal epithelium, a major advantage over conventional eye drops. In murine keratitis models, one single injection of ANHD completely eliminated multidrug-resistant *S. epidermidis* infection, alleviated inflammatory response, and accelerated corneal regeneration (Fig. 7C) [116]. Another study developed frozen reinforced microneedles (FR-MNs) loaded with FeTAP nanozymes. These nanozymes catalyze hydrogen peroxide decomposition to produce oxidative radicals for enhanced bactericidal effects, especially under NIR irradiation. The freezing treatment endows microneedles with high mechanical rigidity for tissue penetration, whereas post-penetration NIR irradiation softens the microneedle matrix to improve adaptability to ocular soft tissues. In a rat ocular infection model, FR-MNs exhibited better therapeutic efficacy than conventional eye drops (Fig. 7D) [117]. These strategies offer promising alternatives to traditional eye drop treatments, providing more efficient, long-lasting, and minimally invasive solutions for ocular infections.

**Table 10.** Summary of representative nanozymes for the treatment of superficial and deep-seated bacterial infections

	Disease types	Bacteria	Modes of administration	Ref.
Superficial infection	Epidermal infection	MRSA; *P. aeruginosa*; *E. coli* and *S. aureus*	Subcutaneous injection; bandage treatment	[98,196]
	Dental caries	*S. mutans*	Using a custom-made applicator	[111]
	Bacterial vaginosis	*G. vaginalis*	Vaginal injection	[115]
	Medical devices	Urinary catheter model	Coat urinary catheters with nanozyme	[34]
	Marine biofouling	Ship model	Paint stainless-steel plates with nanozyme	[28]
Deep infection	Subcutaneous abscess	MRSA; *E. coli*, *P. aeruginosa*	Orthotopic injection	[33,99]
	Sepsis	*E. coli*	Peritoneal injection	[122]
	Pneumonia	MRSA; *E. coli*	Intratracheal instillation; tail vein injection	[99,120]
	Gastritis	*H. pylori*	Intragastric administration	[121]
	Enteritis	*S. typhimurium*	Intragastric administration	[62]
	Keratitis	*P. aeruginosa*	Eye drops	[125]

Superficial infections are mild localized disorders, meaning drug criteria for them are less strict than those for deep infections. Hence, nanozymes with robust catalytic antibacterial capacity are eligible for superficial microbial infection therapy despite their relatively large particle size and weak cytotoxicity.

#### Deep infections

Nanozymes not only exhibit excellent therapeutic effects for superficial bacterial infections but also possess immense therapeutic potential for the treatment of deep-seated bacterial infections in the body (Table 10) [118]. For example, they have demonstrated broad application prospects in the treatment of infections such as MRSA-induced pneumonia [119,120], *H. pylori*-induced gastritis [96,123], *Salmonella*-induced enteritis, sepsis [124], and bacterial contamination of implanted medical devices.

**Fig. 8.**
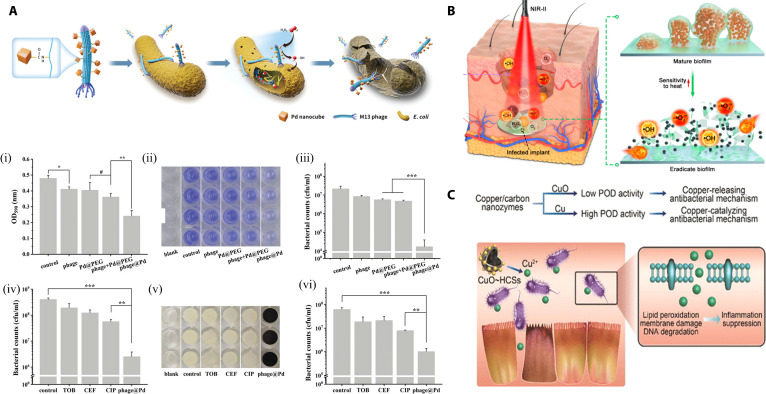
Application of nanozymes in deep infections. (A) Schematic illustration of the structure of phage@Pd and its antimicrobial effect. (i) Quantitative analysis of the biomass of biofilms by ultraviolet absorption at 590 nm (*n* = 4). (ii): Qualitative analysis of the biomass of biofilms by crystal violet staining (*n* = 4). (iii) Quantification of bacteria in biofilm (*n* = 3). (iv) Antibacterial ability of phage@Pd and antibiotics in mature *E. coli* biofilm (*n* = 3). (v) Digital images of *E. coli* biofilm after different treatments. (vi) Antibacterial ability of phage@Pd and antibiotics against bacteria in mature multibacterial biofilm (*n* = 3) [99]. Copyright 2023, Wiley-VCH GmbH. (B) NIR-II light-responsive and triple-enzyme-mimicking CuNx-CNS SAzyme for antibacterial therapies from superficial to deep tissue infections. Copyright 2023, American Association for the Advancement of Science (AAAS) and China Association for Science and Technology (CAST) [123]. (C) Copper/carbon nanozymes exhibited copper state-dependent POD-like activity [62]. Copyright 2023, American Chemical Society.

Bacterial biofilms predominantly develop during chronic bacterial pneumonia, which prevents conventional antibiotics from penetrating and clearing embedded bacteria. Mao and colleagues [99] fabricated phage@Pd nanozymes (Fig. 8A). The bacteriophages within this nanozyme system possess inherent biofilm-targeting capability, enabling phage@Pd to penetrate the surface layer of biofilms. The acidic microenvironment with high H_2_O_2_ concentration inside biofilms activates POD-like activity of the nanozyme, which generates ·OH in situ and thereby efficiently eliminates bacteria (bactericidal efficacy of 96.7%). Similarly, Geng and colleagues [123] synthesized CuN_4_-CNS (central nervous system) nanozymes. Owing to their 2D sheet structure, the material can penetrate biofilms. Its prominent POD- and OXD-like activities catalyze abundant ROS production. Combined with NIR irradiation, this nanozyme can ultimately eradicate deep-seated biofilm infections effectively (Fig. 8B). *H. pylori* is a microaerophilic bacterium that infects approximately half of the global population and serves as a major causative agent of gastritis, gastric ulcers, and gastric cancer. Conventional triple antibiotic therapy is hampered by 3 critical drawbacks: emerging multidrug resistance, premature drug degradation by gastric acid, and dysbiosis of beneficial intestinal flora. To address these limitations, Xi and colleagues [121] fabricated FPB-Co-Ch NP nanozymes. Benefiting from intrinsic SOD- and CAT-mimicking catalytic activities, the nanozymes continuously generate abundant oxygen, and the resultant hyperoxic microenvironment suppresses the metabolism of *H. pylori* and eventually triggers bacterial suffocation. Acute bacterial enteritis can trigger systemic inflammatory responses and may develop into life-threatening sepsis under severe conditions. Gao’s group [62] synthesized 2 kinds of nanozymes, namely, zero-valent copper–carbon nanozyme (Cu^0^@C) and copper oxide–carbon nanozyme (CuO@C). Cu^0^@C displays outstanding POD-like activity, while CuO@C can release abundant Cu^2+^ ions. In the in vivo therapy against acute bacterial enteritis, the intestinal tissues of mice in the CuO-HCSs group showed negligible congestion and hemorrhage, and the load of *Salmonella typhimurium* was drastically reduced after CuO-HCS administration (Fig. 8C).

Medical devices are now widely used. However, their use faces severe challenges related to the risk of device-associated infections. Among these, catheter-associated urinary tract infections (CAUTIs) arise when catheters contaminated with microorganisms introduce pathogens into the urinary tract, thereby triggering the development of the infection. As a result, the sterility requirements for medical devices, especially catheters, have become more stringent. PAA-Cnp, which possesses phospholipase activity, is used as a surface coating agent on the inner surface of catheters. Compared to uncoated catheters, bacterial colonization is reduced, as it hydrolyzes the bacterial cell membrane, preventing bacterial adhesion and deposition on the surface. Such a coating demonstrates potential value in preventing bacterial colonization and biofilm formation on catheter surfaces [34]. Furthermore, studies have shown that nanozymes producing surface-bound ROS can selectively kill bacteria on mammalian cells due to their surface-binding properties and the unexpected detoxification effect of endocytosis. In a mouse model of *P. aeruginosa* PAO1-infected wounds, catheters coated with the nanozyme AgPd0.38 were inserted into pre-infected wounds. After 5 d, no biofilm formation was observed, demonstrating the highly efficient antifouling and antibacterial capabilities of the nanozyme [124]. Keratitis caused by *P. aeruginosa* [especially multidrug-resistant (MDR) strains] is characterized by acute suppurative inflammation with severe tissue destruction, which can rapidly lead to corneal perforation and blindness. However, conventional antibiotics are prone to induce drug resistance, resulting in limited therapeutic efficacy. Moreover, the corneal tissue is highly sensitive, requiring therapeutic agents to possess low toxicity and non-irritation to avoid damaging epithelial cells. Wang et al. [125] designed and synthesized a Co_3_O_4_/Ag hydrogel. This hydrogel nanozyme exhibits high antibacterial activity and biocompatibility, showing excellent bactericidal performance against a variety of bacteria. The high adhesiveness of the hydrogel enhances ocular retention time, while the sustained release of nanozymes enables long-acting antibacterial effects, thereby addressing the problem of low bioavailability associated with conventional eye drops.

Treatment strategies for deep-seated infections vary according to lesion locations, including nasal instillation, intragastric administration, and intraperitoneal injection. These diverse delivery routes impose strict requirements on antibacterial nanozymes, which should possess small particle dimensions, favorable biocompatibility, and microenvironment-responsive catalytic activity. Accordingly, translational practicability must be fully considered during the rational design and synthesis of antibacterial nanozymes.

### Nanozyme treatment for fungal infections

Fungi exhibit strong intrinsic drug tolerance and pose severe global public health threats. The WHO has classified pathogenic fungi represented by *Candida albicans* as top-priority microorganisms endangering human health worldwide. Four major classes of antifungal medications are currently used in clinical practice: polyenes, the pyrimidine analog 5-fluorocytosine, azoles, and echinocandins, each acting on unique molecular targets. However, widespread overuse of antifungals has driven the emergence of abundant drug-resistant fungal isolates. Furthermore, the limited arsenal of licensed antifungals, coupled with frequent adverse side effects including hepatotoxicity, nephrotoxicity, neurotoxicity, allergic responses, and gastrointestinal irritation, renders fungal infections a growing clinical therapeutic challenge.

Recently, nanozymes have emerged as a promising option for addressing fungal infections. Compared with bacteria, fungi possess distinct biological barriers, including rigid cell walls composed of chitin, β-glucans, and mannans, ergosterol-containing membranes, hyphal growth forms, and highly tolerant fungal biofilms. Therefore, antifungal nanozyme design should not simply follow antibacterial ROS-generating strategies. Instead, effective antifungal nanozymes may target multiple fungal-specific structures and processes, including cell wall docking and disruption, ergosterol-associated membrane damage, lipid peroxidation, fungal biofilm penetration, ferroptosis-like fungal death, and microenvironment remodeling. These features are particularly important for broad-spectrum and drug-resistant fungal infections, where conventional antifungal agents often fail owing to limited drug classes and emerging resistance.

**Fig. 9.**
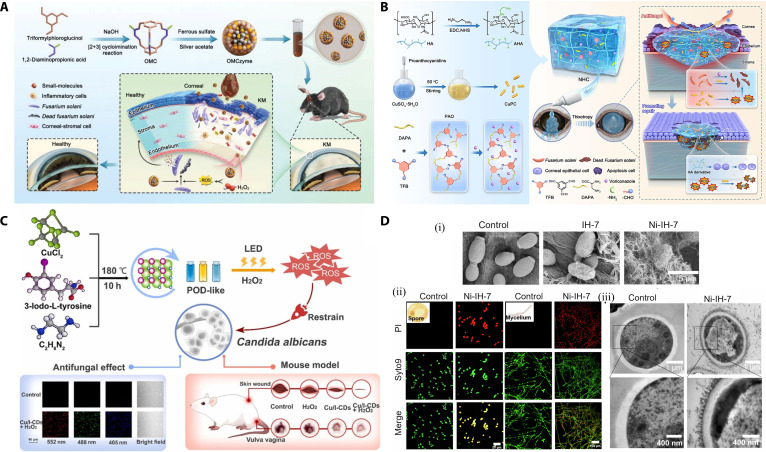
Application of nanozymes in antifungal therapy. (A) Preparation of ionic OMCzymes for the treatment of keratomycosis [126]. Copyright 2024, Wiley-VCH. (B) Synthesis of NHC for the treatment of fungal keratitis [127]. Copyright 2024, Elsevier. (C) Synthesis of Cu/I-CD nanozymes and antifungal action against *C. albicans* infection [128]. Copyright 2023, Elsevier. (D) (i) SEM characterization of *C. albicans* treated with Ni-IH-7 nanotubes. (ii) Confocal microscopy of planktonic yeast and mycelium biofilm of *C. albicans* treated by Ni-IH-7 nanotubes using live (Syto9)/dead [propidium iodide (PI)] staining. (iii) SEM and TEM characterization of *C. albicans* treated with Ni-IH-7 nanotubes [129]. Copyright 2024, Springer Nature.

Studies have indicated that OMCzyme exhibits POD-like activity, enabling it to convert H_2_O_2_ into ·OH. The fungal cell wall facilitates the penetration of ·OH, leading to potential damage and induced cell death in *Fusarium solani*. This nanozyme demonstrates targeted antifungal activity, capable of penetrating the corneal stroma and efficiently eradicating *F. solani* while displaying minimal cytotoxicity toward human corneal epithelial cells, thereby offering a promising therapeutic strategy for fungal keratitis (Fig. 9A) [126]. Furthermore, a nanohybrid hydrogel coating (NHC) consisting of voriconazole and multi-enzyme-mimetic copper-proanthocyanidin (CuPC) nanozymes has been developed to treat *F. solani*-induced keratitis. This system promotes cell proliferation via hyaluronic acid derivatives while cooperatively scavenging ROS via the POD-, CAT-, and SOD-like functions of CuPC (Fig. 9B) [127]. Studies have demonstrated that copper and iodine-doped CDs (Cu/I-CDs) possess POD-like activity, generating superoxide anions (·O_2_^−^) and hydroxyl radicals (·OH), which exert a fungicidal effect against *C. albicans* biofilms (Fig. 9C) [128]. In a similar manner, multifunctional gold nanozymes carrying AmB target fungal membrane ergosterol, inducing pore formation and loss of membrane integrity. By mimicking POD-like activity, they catalyze H_2_O_2_ to produce ·O_2_^−^, destroying cellular structures and treating *C. albicans* infections. Yuan et al. [129] developed peptide nanozymes that mimic the mechanism of action of antimicrobial peptides (AMPs) and antimicrobial enzymes (AMEs) through de novo design and peptide assembly. The resulting Ni-IH-7 nanotubes exhibit phospholipase C- and POD-like activities facilitated by Ni coordination. The assembled nanotubes exhibit cascade antifungal effects, including outer mannan docking, cell wall disruption, lipid peroxidation, and subsequent ferroptotic cell death, synergistically eliminating over 90% of fluconazole-resistant *C. albicans* within 10 min on a disinfection pad. Live/dead staining, scanning electron microscopy (SEM), and transmission electron microscopy (TEM) results visually demonstrate the excellent antifungal capacity of Ni-IH-7 nanozyme (Fig. 9D). Iron oxide NPs (IONPs) adjust the frequency and spatial distribution of electromagnetic fields to selectively bind and interact with fungi, thereby promoting the localized accumulation of nanozymes and the targeted in situ killing effect of ROS. Additionally, by mimicking POD-like activity, IONPs can activate H_2_O_2_ to generate ROS, producing a potent biofilm-killing effect [130]. Encouragingly, the Ce-MOF nanozyme synthesized by Sharmouk and colleagues [131] exhibits broad-spectrum antifungal activity. Colony-forming unit (CFU) assays reveal that the nanozyme achieves an antifungal efficiency of 93.3% to 99.3% against 5 pathogenic fungi, namely, *Aspergillus flavus*, *Aspergillus niger*, *Aspergillus terreus*, *C. albicans*, and *Rhodotorula glutinis*.

However, current research on antifungal nanozymes still has much room for expansion, and most existing antifungal evaluations center on *C. albicans* rather than a full spectrum of pathogenic fungi. Follow-up work ought to construct targeted nanozymes active against broad-spectrum fungal strains, offering more potent therapeutic options to combat refractory fungal infectious diseases.

### Nanozyme therapy for viral infections

Currently, the prevention and control of viral diseases mainly consist of 2 major approaches: vaccination and treatment with antiviral drugs and antibodies. The development of antiviral drugs follows 2 core strategies: targeting the virus itself and targeting host cells. Strategies against the virus include targeting viral envelopes [123], viral surface proteins [132], and viral nucleic acids [133]. Host cell-targeted strategies include but are not limited to interfering with viral entry pathways [134], regulating intracellular redox homeostasis [135], and modulating immune cells [136]. Existing antiviral therapeutics are categorized into 7 types: neutralizing antibodies, recombinant soluble human neutralizing receptors, antiviral CRISPR/Cas systems, interferons, antiviral peptides, antiviral nucleic acid polymers, and small-molecule antiviral agents. In addition, proteolysis-targeting chimera (PROTAC) technology has recently been explored for antiviral therapy by recruiting E3 ligases to degrade viral or virus-related host proteins. Although these strategies are distinct from nanozyme catalysis, they highlight the increasing importance of target-specific and host-directed antiviral interventions.

Natural enzymes possess intrinsic capabilities to recognize and disrupt viral envelopes, thereby exerting potent antiviral activity. For example, esterases CbAE-1 and CbAE-2 act against dengue virus (DENV), Zika virus (ZIKV), severe acute respiratory syndrome coronavirus 2 (SARS-CoV-2), human immunodeficiency virus (HIV), and herpes simplex virus (HSV); lipoprotein lipase (LPL), hepatic triglyceride lipase (HTGL), and LPL-Ps act against hepatitis C virus (HCV); secretory phospholipase A₂ (sPLA2) acts against DENV, other enveloped viruses, and yellow fever virus; phospholipase CM-II-sPLA2 targets enveloped viruses from the endoplasmic reticulum membrane, such as HCV, DENV, and Japanese encephalitis virus (JEV); phospholipase sPLA2-X acts against lentiviruses, etc.

Compared with antibacterial nanozyme research, antiviral nanozyme studies remain less developed, but they provide a distinct opportunity to apply catalytic materials to viral inactivation, host redox regulation, and immune-adjuvant design. Based on current evidence, antiviral nanozymes can be broadly divided into 3 mechanistic categories. The first category directly targets viral structures, especially lipid envelopes and capsid or coat proteins, through lipid peroxidation, oxidative protein damage, or catalytic hydrolysis. The second category regulates the redox state of infected host cells to suppress viral replication or reactivation. The third category functions as catalytic immune adjuvants by promoting dendritic cell maturation, antigen presentation, and mucosal or systemic adaptive immunity. Organizing antiviral nanozymes according to these targets helps distinguish direct virucidal activity from host-directed antiviral modulation and immunoprotection.

**Fig. 10.**
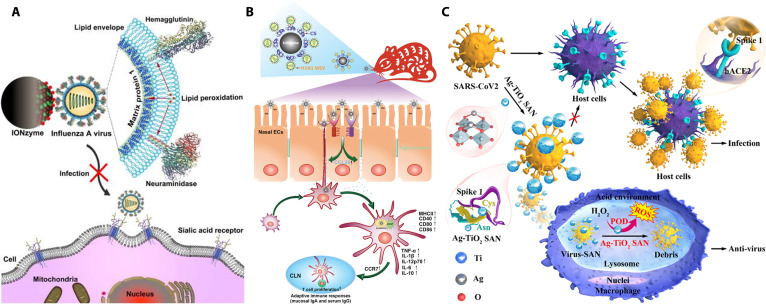
Application of nanozymes in antiviral therapy. (A) Schematic of viral lipid peroxidation by IONzymes for virus inactivation [137]. Copyright 2019, Ivyspring International Publisher. (B) Schematic of proposed mechanism for enhancing antigen-specific immune response by the CS-IONzyme-based influenza vaccine [138]. Copyright 2020, Wiley-VCH. (C) Schematic of Ag-TiO_2_ SAN with anti-SARS-CoV-2 activity [139]. Copyright 2021, Elsevier.

For example, IONzymes directly interact with IAV (influenza A virus) particles to enhance lipid peroxidation, disrupt the viral lipid envelope, and generate free radicals. These effects further damage adjacent viral proteins (including hemagglutinin, neuraminidase, and matrix protein 1), thereby impairing multiple viral structures and functions, inhibiting host cell infection, and ultimately achieving viral inactivation. When incorporated into masks, IONzymes improve the mask’s protective efficacy against 3 key virus subtypes: H1N1, H5N1, and H7N9 (Fig. 10A) [137]. Chitosan-modified iron oxide nanozymes (CS-IONzyme), with POD-like activity, can serve as vaccine adjuvants to inactivate viruses and enhance immune responses against influenza virus A subtype (IVAS)-H1N1 (Fig. 10B). The nanozyme can modulate intracellular ROS level and activate the TLR2/4 signaling pathway, which in turn stimulates CCL20 secretion. The released CCL20 recruits DCs and promotes their maturation, greatly enhancing antigen uptake and presentation. This cascade potently activates both mucosal and systemic adaptive immunity, driving abundant production of influenza-specific secretory immunoglobulin A (IgA) and IgG. These antibodies build dual immune defenses in the respiratory tract and throughout the body, ultimately protecting the host from influenza virus infection [138]. TiO_2_-supported single-atom Ag nanozymes (Ag-TiO_2_ SAN) exhibit high POD-like activity, generate ROS under acidic conditions, and exert effective antiviral activity against SARS-CoV-2 (Fig. 10C) [139]. V_2_O_5_ nanosheets exhibit GPx-like activity [135], counteract cellular ROS induced by HIV-1 infection, and suppress HIV-1 reactivation and replication. With their lipoxidase (LOX)-mimicking activity, bimetallic iron nanomaterials (Fe_2_ diatomic catalyst) [140] efficiently compromise the influenza viral envelope through lipid peroxidation, leading to inactivation of various influenza strains such as H1N1 and H9N2. Their antiviral performance surpasses that of natural LOX. Furthermore, Fe_2_ DAC is suitable for incorporation into air purifier filter replacements to eliminate influenza viruses from the air.

## Summary and Outlooks

The central message of this Review is that the antimicrobial performance of nanozymes is governed not only by their intrinsic catalytic activity but also by the coordinated interplay among structure–activity relationships, infection microenvironment constraints, and engineering-enhanced synergistic strategies. Inspired by natural enzyme-mediated host defense, antimicrobial nanozymes have evolved from simple enzyme mimics into multifunctional catalytic systems capable of integrating catalytic regulation, microenvironment responsiveness, targeted delivery, and multimodal therapeutic strategies. Throughout this Review, we emphasize that rational antimicrobial nanozyme design should not rely solely on material composition or catalytic activity. Instead, effective antimicrobial performance arises from the integration of bioinspired catalytic motifs, structural optimization, infection microenvironment adaptation, and application-driven engineering. This structure–activity-guided design framework provides a more comprehensive understanding of nanozyme-mediated antimicrobial therapy and offers practical guidance for the development of next-generation antimicrobial nanozymes. Nevertheless, although nanozymes display attractive prospects for antimicrobial treatment, multiple obstacles still hinder their large-scale clinical translation. In Table 11, we summarized major translational challenges and corresponding design solutions.

**Table 11.** Major translational challenges and proposed solutions for antimicrobial nanozymes

Challenge	Proposed solution	Design rationale
Long-term accumulation	Biodegradable, metabolizable, or renal-clearable nanozymes	Reduce organ retention and chronic toxicity
Heavy-metal toxicity	Low-metal, metal-free, or self-limiting catalytic systems	Minimize ion leakage and off-target oxidative injury
Poor infection-site accumulation	Hydrogels, microneedles, coatings, eye drops, wound dressings	Increase local retention and reduce systemic exposure
Low H_2_O_2_/weak acidity	GOx cascade, H_2_O_2_ self-supply, pH-amplifying systems	Maintain POD/Fenton-like catalysis
Hypoxia	CAT-like oxygen generation, type I PDT, oxygen carriers	Improve oxygen-dependent catalysis and PDT
High GSH/antioxidant buffering	GSH depletion, redox cascade, localized ROS generation	Overcome antioxidant defense
Biofilm barrier	DNase-like, phospholipase-like, hydrolase-like, targeting nanozymes	Degrade EPS and expose embedded bacteria
Resistance/tolerance	Multimodal therapy, nanozyme–antibiotic combinations, phage or immune synergy	Avoid sublethal exposure and adaptive compensation
Benchmarking inconsistency	Standardized reporting checklist	Improve cross-study comparability

1. Long-term biosafety, biodistribution, and metabolic fate. Current antimicrobial nanozyme studies still rely heavily on in vitro assays, short-term animal models, and superficial infection settings, whereas systematic long-term biosafety evaluation remains insufficient. For in vivo therapeutic use, several issues require rigorous analysis, including biodistribution, pharmacokinetics, biodegradation, metabolic clearance, protein–corona formation, immunogenicity, organ accumulation, repeated-dose toxicity, microbiome disturbance, and potential chronic toxicity. These concerns are particularly important for heavy metal-based and ROS-generating nanozymes, because uncontrolled metal ion release, persistent oxidative stress, hepatic or splenic retention, and excessive inflammatory activation may cause off-target tissue damage. Therefore, translational evaluation should not only assess short-term bacterial killing but also determine whether catalytic activity persists after protein–corona formation, whether released ions or degradation products accumulate in organs, and whether repeated exposure causes chronic inflammation or immune dysregulation.

To address these safety challenges, future antimicrobial nanozyme design should prioritize biodegradable, metabolizable, or renal-clearable materials, minimize toxic metal components, and incorporate self-limiting or infection-triggered catalytic activity. Local delivery platforms, including hydrogels, wound dressings, microneedles, coatings, eye drops, and implant surfaces, may reduce systemic exposure while increasing local antimicrobial efficacy. In addition, combining nanozymes with antibiotics, phages, immunotherapy, or other catalytic systems may lower the required nanozyme dose and thereby reduce long-term toxicological risks.

2. Resistance evolution and adaptive tolerance. Nanozyme-mediated antimicrobial therapy may reduce the likelihood of classical target-specific resistance because it often relies on multi-target physicochemical damage, including oxidative stress, membrane disruption, metal ion interference, metabolic perturbation, and immune-mediated clearance. However, it should not be assumed that nanozymes are free from resistance risk. Prolonged sublethal exposure to ROS-generating nanozymes may induce reversible antioxidant tolerance or stable adaptive resistance by up-regulating bacterial antioxidant pathways, including SOD, CAT, POD, and GSH-related systems. Bacteria may also reduce nanozyme susceptibility through biofilm thickening, altered surface charge, efflux regulation, metabolic dormancy, or stress-response remodeling.

Therefore, resistance evolution should become an important evaluation component in antimicrobial nanozyme research. Serial-passage assays, sublethal exposure models, recurrent infection models, and microbiome-level analyses are needed to determine whether nanozyme treatment promotes adaptive tolerance or long-term resistance. To reduce this risk, emerging strategies include multimodal synergistic therapy, cascade catalytic systems, nanozyme–antibiotic combinations, photothermal or sonodynamic assistance, immune activation, and infection-triggered catalytic amplification. These approaches may increase local effective killing, avoid persistent subinhibitory exposure, and suppress bacterial adaptive compensation.

3. Standardized antimicrobial evaluation and performance benchmarking. The absence of unified and standardized evaluation frameworks is a critical challenge, which severely impedes the horizontal performance comparison and objective benchmarking of diverse nanozyme systems. First, nanozyme treatment concentrations vary substantially across published studies, and there is no universally recognized criterion for the quantitative definition and calibration of nanozyme catalytic activity in academia. Second, key experimental variables lack standardized regulation. Existing studies adopt inconsistently labeled bacterial strains and disparate initial inoculation optical density (OD) values. No unified reference protocols are available for culture medium formulations, reaction solvents, buffer pH values, core lesion microenvironmental parameters (e.g., oxygen and hydrogen peroxide levels), and bacterial incubation durations. Moreover, most studies fail to set up adequate parallel control groups to systematically compare the antibacterial efficacy against planktonic bacteria and biofilm-embedded bacteria. Third, the detection methodologies for antibacterial performance remain fragmented. Uniform specifications are lacking for routine quantitative assays, including OD growth curve monitoring, plate colony counting, as well as MIC and MBC measurements. Fourth, standardized evaluation criteria for anti-biofilm performance are not yet established. Quantitative detection approaches for total biofilm biomass, extracellular polymeric matrix content, deep-layer viable bacteria, and persister cell clearance differ among different reports. Such inconsistent experimental parameters and disjointed evaluation paradigms greatly hinder the objective cross-study comparison of nanozyme antibacterial and anti-biofilm performance. Therefore, it is urgent to establish comprehensive, reproducible, and standardized testing protocols to promote the standardized development of antibacterial nanozyme research.

4. Stability. The catalytic activity, structural stability, and enzymatic efficiency of nanozymes may change in physiological environments (such as pH, temperature, and ionic strength), thus affecting their antimicrobial effects. Therefore, optimizing the stability of nanozymes, enhancing their activity in different biological environments, and ensuring their persistence in the complex in vivo environment have become key issues in current research. Designing tailored nanozymes with environmental tolerance or responsiveness according to variable physiological conditions across diverse application scenarios constitutes one effective strategy to accelerate the industrialization of nanozymes. In addition, surface modification can be applied to nanozymes to isolate them from complex environments and maintain stable catalytic activity. Alternatively, heteroatom doping is adopted to improve their structural rigidity and environmental adaptability.

5. Target specificity. the pro-oxidation treatment of nanozyme is usually carried out by the indiscriminate production of ROS for antibacterial, antitumor, and other treatments, which will undoubtedly also cause damage to normal cells and tissues, so it is necessary to build specific nanozyme. It enables accurate biometrics, reduces nonspecific responses, improves therapeutic effectiveness, and enhances biocompatibility. At present, the main targeting strategies are as follows. First, microenvironment-responsive targeting, represented by pH-responsive nanozymes: Such nanozymes remain low catalytic activity under normal physiological pH, while their catalytic activity increases dramatically once they reach acidic lesion sites, thereby reducing damage to normal tissues. Second, bacterial-targeted ligands: Specific recognition molecules such as lysozyme and lectins are modified on the surface of nanozymes to achieve bacterial targeting. Third, magnetic targeting: Magnetic nanozymes can be directionally enriched at infected sites under the guidance of an external magnetic field. Fourth, bacterial membrane coating: It realizes homologous targeting and immune evasion, and enables precise recognition of homologous pathogenic bacteria.

6. Large-scale synthesis. Currently, the research and application of nanozymes are largely confined to small-scale laboratory production. The activity of nanozymes is influenced by factors such as particle size, surface charge, shape, functional group modifications, and so on. During scale-up production, differences in the microenvironment surrounding reactants, such as concentration and temperature, compared to small-scale production, could indeed impact the enzyme-like activity of nanozymes and consequently affect their functional performance. Moreover, many nanozymes are produced involving noble metals such as Au, Pd, and Ru, and often require a high-temperature and high-pressure process. These production methods greatly escalate production costs, thereby limiting the clinical applications of such nanozymes. Therefore, developing more efficient, environmentally friendly, and low-cost synthesis routes is essential. To address the above issues, establishing standardized production procedures, replacing noble metals with abundant metals such as iron, manganese, copper, and cobalt, and developing synthetic routes under room temperature and atmospheric pressure are essential approaches to realize large-scale production.

7. Low-temperature catalysis. It should be another important application direction for nanozyme catalysis. Viruses maintain high activity in low-temperature environments, accelerating their spread during cold chain transportation. Traditional disinfection systems face limitations in antiviral efficiency at low temperatures, e.g., −10 °C, requiring several times higher disinfectant concentrations to eliminate viruses completely. However, high-concentration disinfectants not only pose safety hazards to food but also increase cold chain transportation costs. Developing catalytic systems that exhibit efficient performance under low-temperature conditions to eradicate pathogenic agents is thus both necessary and promising.
